# Computational Approaches for Decoding Select Odorant-Olfactory Receptor Interactions Using Mini-Virtual Screening

**DOI:** 10.1371/journal.pone.0131077

**Published:** 2015-07-29

**Authors:** K. Harini, Ramanathan Sowdhamini

**Affiliations:** National Centre for Biological Sciences (TIFR), GKVK Campus, Bellary Road, Bangalore, India; Duke University, UNITED STATES

## Abstract

Olfactory receptors (ORs) belong to the class A G-Protein Coupled Receptor superfamily of proteins. Unlike G-Protein Coupled Receptors, ORs exhibit a combinatorial response to odors/ligands. ORs display an affinity towards a range of odor molecules rather than binding to a specific set of ligands and conversely a single odorant molecule may bind to a number of olfactory receptors with varying affinities. The diversity in odor recognition is linked to the highly variable transmembrane domains of these receptors. The purpose of this study is to decode the odor-olfactory receptor interactions using *in silico* docking studies. In this study, a ligand (odor molecules) dataset of 125 molecules was used to carry out *in silico* docking using the GLIDE docking tool (SCHRODINGER Inc Pvt LTD). Previous studies, with smaller datasets of ligands, have shown that orthologous olfactory receptors respond to similarly-tuned ligands, but are dramatically different in their efficacy and potency. Ligand docking results were applied on homologous pairs (with varying sequence identity) of ORs from human and mouse genomes and ligand binding residues and the ligand profile differed among such related olfactory receptor sequences. This study revealed that homologous sequences with high sequence identity need not bind to the same/ similar ligand with a given affinity. A ligand profile has been obtained for each of the 20 receptors in this analysis which will be useful for expression and mutation studies on these receptors.

## Introduction

The sense of smell has been the least understood of all the five human senses known till recent times. The detection of odorants is essential for survival of an individual. The discriminatory power of olfactory receptors (ORs) is such that it can perceive thousands of volatile chemicals as having different odors. It is known that the olfactory system uses a combinatorial receptor coding scheme to decipher the odor molecules. One OR can recognize multiple odorants and one odorant is recognized by multiple ORs [1]. A slight structural change in the odorant or a change in the concentration of the odorant in the environment results in a change in the odor-code of these receptors.

Each mammalian olfactory receptor neuron encodes only one OR [2–4]. The axons of the neurons expressing the same olfactory receptor converge to one olfactory bulb, which then processes the information to the brain [5]. ORs are structurally similar to G-Protein Coupled Receptors (GPCRs) and contain seven transmembrane (TM) domains connected by loops. The functionally important residues are present on the transmembrane helices 2–7 [6–8]. In insects, the detection of odorants is performed by a smaller set of about sixty odorant receptors [9]. Due to the lack of X-ray crystal structures of olfactory receptors and the difficulties in heterologous expression of ORs, very few ORs have been “de-orphaned” *i*.*e*. associated with their ligands (odors). Odorant-OR binding studies are limited to a small number of ORs that can be tested at one condition. The number and mixture of odorants that can be used in a single study are also limited.

Odor molecules belong to a variety of chemical classes: from alcohols, aldehydes, ketones and carboxylic acids to sulphur-containing compounds and essential oils. The physicochemical descriptors of odor molecules play an important role in the prediction of odor response by the OR [10] [11]. Very identical OR sequences can have a structural bias for ligand specificity on the basis of the number of carbon atoms present in the ligands [12]. About 8000 odorants have been identified in food. KFO (Key Food Odorants) has identified about 400 odorants which have been characterized and this number approximately equals the number of ORs found in humans [13]. The response of mixture of odorants is neither the additive nor an average of its components [14]. Mixing some odorants lead to the emergence of novel perceptual qualities that were not present in each component, suggesting that odorant mixture interactions occurred at some levels in the olfactory system [15]. Odorant molecules in a mixture could act as an antagonist and hinder the response of the receptor to agonists. Thus, deciphering the complex coding mechanisms requires large scale analysis to compare and consolidate odorant-OR interaction across several receptors.

Molecular docking, an *in silico* approach, can be used to model the interaction between a small molecule and a protein at atomic levels. This method allows us to characterize the binding properties of the small molecule to the receptor and the discriminatory mechanisms, as well as helping to elucidate fundamental biological processes [16]. Docking involves two steps—predicting of binding conformation of the ligand, and predicting the binding affinity of the ligand to the receptor. Knowing the location of the binding site increases the efficiency of the docking tool. This information about the binding site can be obtained from experimental and mutational data. The earliest method of docking assumed a lock-and-key model for ligand-receptor interaction [17]. Since the functional protein is actively re-shaped, the induced fit theory of protein-ligand docking was used to induce flexibility to both receptor and ligand which would result in an accurate prediction of their interactions [18]. At a large scale, docking tools help analyze the interactions of receptors to a large set of ligands, and in scoring the best ligand out of the set. Several *in silico* docking tools have been developed in the recent past, which helps us analyse protein-ligand interactions [19–26].

One of the major challenges in the field of docking is handling the flexibility of protein receptors efficiently. Proteins are in constant motion between different conformational states with similar energies and this fact is still disregarded in many docking studies due to the large computational time required and the inherent limitations of such methods to sample alternate conformations accurately. The use of an ensemble of protein conformations as a starting point helps to sample various functional states of the receptor protein. The computational time for this approach scales linearly with the number of protein structures that constitute the ensemble [27]. Lack of imparting complete protein flexibility in docking approaches still remains a bottleneck in justifying the outcome of a docking analysis. The X-ray crystallographic structures reveal the buried surface area of a ligand as being between 70 to 100% and thus the binding site orientation can be greatly influenced by protein flexibility and solvation [28]. Inducing flexibility at the ligand binding site can lead to the sampling of a wide range of ligands, instead of discarding them at the initial stages of docking as non-binders. The scoring functions that accompany a docking tool might be simplified to compromise between speed and accuracy. Certain scoring functions tend to provide better scores for certain type of binding sites [29] [30]. This dependence of scoring function on the binding site should be properly weighted. GLIDE [31] appears to be one of the best docking suites and provides most consistent results with respect to diversity of binding site, ligand flexibility and overall sampling. Glidescore (gscore) is an effective scoring function and shows maximum accuracy when compared to other docking tools such as GOLD and ICM [32]. GLIDE provides an opportunity to minimize the receptor in the membrane environment before docking, which proves to be very helpful in the case of membrane bound systems such as ORs.

Computational docking approaches can be useful in understanding odor-receptor interactions, since very few ORs have been de-orphaned experimentally. Of the huge set of mammalian olfactory receptors, 400 in *H*. *sapiens* and 1000 in *M*. *musculus*, only ~50 receptor-odorant interactions are known [8]. The known interactions are based on studies with a limited set of odorants and their mixtures. OR orthologs respond to similar odors with dramatic differences in efficacy and potency even if OR orthologs respond to similar set of odors more frequently than paralogs [7].

This study aims at building an odorant profile for a chosen set of mammalian ORs using a receptor dataset of ten human and mouse homologous pairs of ORs and 125 known odorants as the ligand data set. The analysis helped build an odorant profile for an OR and compare the odorant profiles across homologous ORs. We employed the induced-fit docking (IFD) protocol to obtain the binding energy scores of odorants to the ORs. The odorant profiles for single ORs have been obtained using a limited set of odor molecules earlier [33–38]. The earliest analysis was on mouse OR that responds to eugenol. One study reports the importance of residue Ser 113 as the most important residue required for ligand binding [35]. The analysis on the same OR under different experimental conditions, shows residue Phe 182 to be important in ligand binding. The mutation of this Phe residue results in a loss of response to eugenol [37]. We present a longitudinal study in this paper, where we have developed a reliable computational pipeline to study more than one OR against a large number of ligands. The methodology in this study has been standardised using the binding site information of the mouse eugenol receptor (mOR-EG) and validated using known experimental data, where possible.

## Methodology

### Receptor Dataset

A subset of twenty mammalian olfactory receptors, out of the 100 ORs that were modeled using homology modeling protocol in our previous analysis [39], were used for the analysis to decipher the odorant profile of the twenty ORs. From phylogenetic analysis on human and mouse ORs, ORs are known to form ten distinct clusters [40] (B.Nagarathnam, Ph.D thesis, 2013) which can further be divided into smaller subclusters. One human olfactory receptor was selected from every cluster of human OR phylogeny for this analysis. Thus, ten human ORs obtained were aligned to the 338 mouse OR sequences [40]. The ten mouse ORs, which clustered very close to each of the ten human ORs used in this study, were selected for docking analysis, thereby leading to ten pairs of closely related OR sequences from the human and mouse OR repertoires which were used for unravelling the odorant profiles.

### Ligand Dataset

One hundred and twenty-five odorant molecules were chosen for this study (Table 1). These molecules were selected from earlier studies which have proven them to be odorants that can elicit response from olfactory receptors by *in-vitro* or *in-vivo* analysis [41–45]. The odorants included mammalian and insect-specific odorant molecules. The molecules belonged to different chemical classes like alcohols, ketones, acids, aldehydes and sulphuric compounds. Known antagonists of ORs were also present in the collection of odorants. The three-dimensional coordinates of ligands were obtained from PubChem3D [46] and prepared for docking studies using the Ligprep suite of Schrodinger GLIDE software (**Schrödinger Release 2013–1**: LigPrep, version 2.6, Schrödinger, LLC, New York, NY, 2013).

**Table 1:** List of odorants used in the docking analysis. The odorants have been classified based on their functional groups. Odorants which are known to bind to insect ORs are listed separately. There are two odorants specific to ORs from model organisms *C*. *elegans* (odr-10) and *M*. *musculus* (mOR-EG). Repellents were chosen for clustering along with the odorants (based on chemical similarity) to understand their similarity to the odorants.

Odorant	Pubchem ID
**ALCOHOLS**	
**1-hexanol**	8103
**2-ethyl 1-hexanol**	7720
**1-heptanol**	8192
**1-octanol**	957
**2-octanol**	20083
**3-octanol**	11527
**4-octanol**	11515
**1-nonanol**	8914
**2-nonanlol**	12367
**1-decanol**	8174
**1-dodecanol**	8193
**Geraniol**	637566
**Phenyl methanol**	244
**Menthol**	16666
**Thymol**	6989
**Gvaiacol**	460
**Maltol**	8369
**ACIDS**	
**propionic acid**	1032
**isobutyric acid**	6590
**butyric acid**	264
**hexanoic acid**	8892
**heptanoic acid**	8094
**octanoic acid**	379
**nonanoic acid**	8158
**decanoic acid**	2969
**dodecanoic acid**	3893
**isovaleric acid**	10430
**Trans-cinnamic acid**	444539
**Pyrazine**	9261
**2-methyl pyrazine**	7976
**2-isobutyl-3-methoxypyrazine**	32594
**ALDEHYDES**	
**Hexanal**	6184
**Heptanal**	8130
**Octanal**	454
**Nonanal**	31289
**Decanal**	8175
**Undecanal**	8186
**Dodecanal**	8194
**Benzaldehyde**	240
**Lyral**	91604
**+Citronellal**	75427
**-Citronellal**	443157
**Citral**	638011
**Cinnamaldehyde**	637511
**Helional(phenobarbital)**	4763
**Para-anisaldehyde**	31244
**Vanillin**	1183
**Ethyl-vanillin**	8467
**KETONES**	
**2-heptanone**	8051
**2-octanone**	8093
**3-octanone**	246728
**2-nonanone**	13187
**3-nonanone**	61235
**2-decanone**	12741
**2-dodecanone**	22556
**3-hydroxybutan-2-one**	179
**6-methyl-5-hepten-2-one**	9862
**piperonyl acetone**	62098
**Menthone**	26447
**Beta-ionone**	638014
**Cyclohexanone**	7967
**Acetophenone**	7410
**Hedione**	102861
**Camphor**	2537
**ESTERS**	
**isoamyl acetate**	31276
**ethyl butyrate**	7762
**ethyl isobutyrate**	7342
**butyl butyrate**	7983
**ethyl hexanoate**	31265
**ethyl heptanoate**	7797
**ethyl octanoate**	7799
**ethyl nonanoate**	31251
**ethyl decanoate**	8048
**methyl hexanoate**	7824
**methyl heptanoate**	7826
**methyl octanoate**	8091
**methyl nonanoate**	15606
**methyl decanoate**	8050
**methyl salicylate**	4133
**geranyl acetate**	1549026
**SULPHOREOUS**	
**dimethyl disulfide**	12232
**3-methyl thiobutrnoate**	409240
**Thiazole**	9256
**Benzothiazol**	7222
**LACTONES**	
**Cumarin**	323
**Gamma-decalactone**	12813
**OTHERS**	
**Pyridine**	1049
**Quinolin**	7047
**Indole**	798
**Anisol**	7519
**Trans-anethol**	637563
**Cineol**	10106
**Estragol**	8815
**Safrol**	5144
**Citralva**	1551246
**Limonene**	22311
**INSECT ODORS**	
**Γ-hexalactone**	12756
**butyric acid**	264
**hexanoic acid**	8892
**Α-terpineol**	17100
**Linalool**	6549
**Acetaldehyde**	177
**Butanal**	261
**Pentanal**	8063
**2-pentanone**	7895
**1-butanol**	263
**1-pentanol**	6276
**2-pentanol**	22386
**3-methyl butanol**	31260
**3-methyl-2-buten-1-ol**	11173
**1-octen-3-ol**	18827
**z-2-hexenol**	5363388
**propyl acetate**	7997
**butyl acetate**	31272
**pentyl acetate**	12348
**hexyl acetate**	8908
**Isobutylacetate**	8038
**E-2-hexenyl acetate**	5363374
**methyl butyrate**	12180
**ethyl-3-hydroxybutyrate**	62572
**ethyl propionate**	7749
**ethyl-trans-2-butenoate**	429065
**diethyl succinate**	31249
**Odorants specific to organisms**	
**Diacetyl (*C*.*elegans*)**	650
**eugenol(*M*.*musculus*)**	3314
**Repellents**	
**DEET**	4284
**Picaridin**	125098
**Eucalypton**	10390702
**Linalool**	6549
**Alpha-thujone**	261491
**Beta-thujone**	249286
**ethyl anthralite**	6877
**methyl N,N,-dimethylanthranilite**	82336
**butyl anthralite**	24433
**methyl p-tert- butyl phenyl acetate**	605629
**ethyl chrysanthemate**	7334
**2-isobutyl 3-methoxy pyrazine**	32594
**Allyl-ionoe**	5365976
**2-sec-butyl-3-methoxypyrazine**	520098

### Grouping of Ligands by Clustering

MOLPRINT2D [47] [48] and Tanimoto co-efficient [49] of the CANVAS module (**Schrödinger Release 2013–1**: version 2.6, Schrödinger, LLC, New York, NY, 2013) in Schrödinger software [50] [51] were used to cluster the ligands based on their molecular and chemical features. The molecular descriptor calculates numerical binary values such as log P, molecular weight, electronic and valence states, 3-D pharmacophore interactions and the distance between molecular fingerprints from their molecular features. The Tanimoto-coefficient calculates the chemical fingerprint of each odorant using fragment-based binary representation. Given two molecules, the method calculates similarity upto a given bond along a linear path. The branching points and cyclic patterns from each of the linear paths are then detected. Using a proprietary hashing method, a given bit number is set for each pattern. Fourteen known repellents [52] were included in the clustering for associating their relationship to known odorant molecules through clustering approach (Table 1). The repellents were not used in the current docking analysis and could form a basis for a future study on comparing the binding patterns of odorants and repellents. The resulting ligand clusters were used to analyse the docking results.

### Induced-Fit Docking

**Fig 1:**
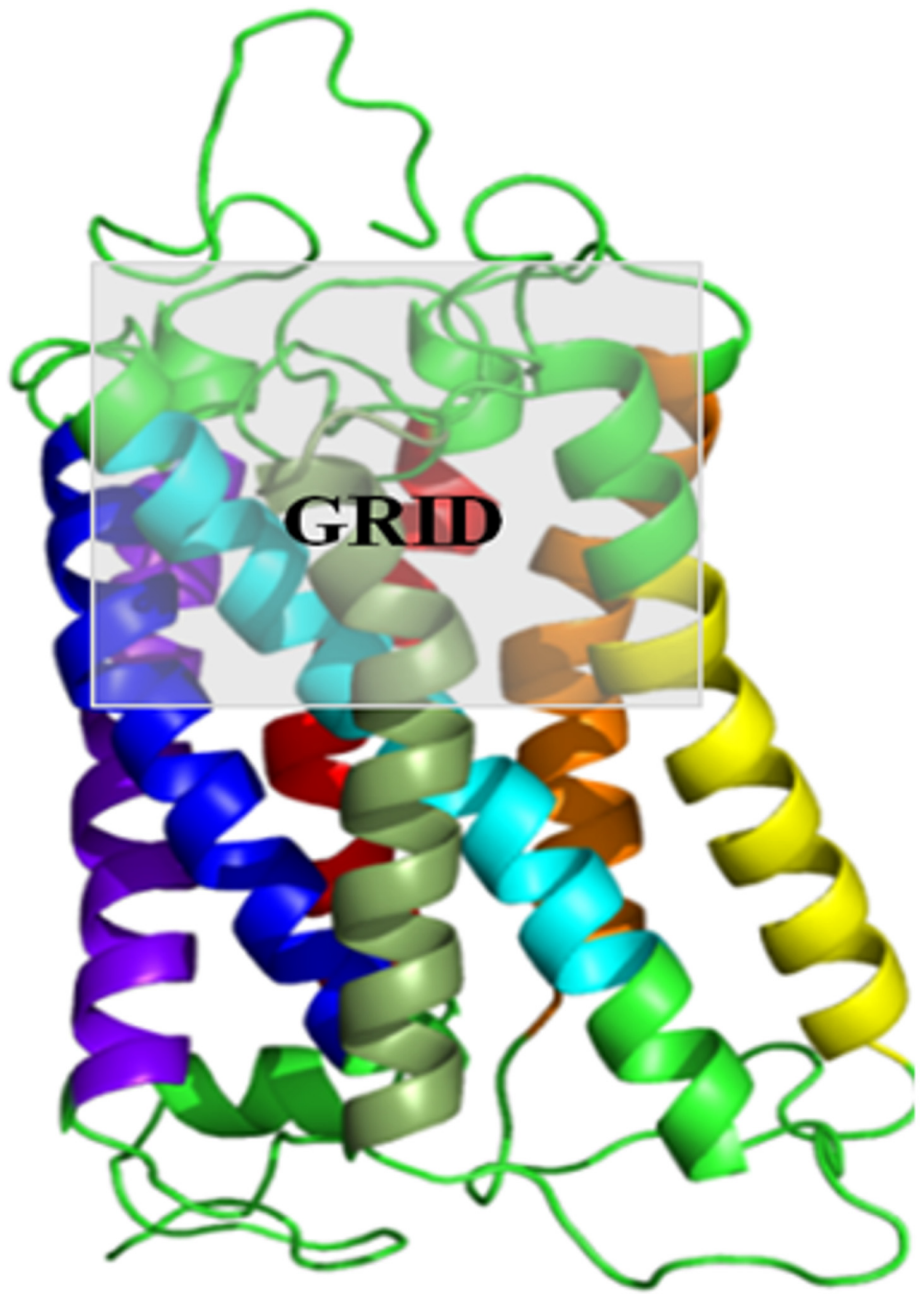
GRID selected for Induced Fit Docking. Induced Fit docking protocol was standardized using the experimental data available on mouse ORs that responds to eugenol (mOR-EG). Different grid parameters and constraints were used to standardize the protocol as shown in Table 2. The use of the upper half of the receptor facing the extracellular milieu gave the best score for eugenol binding as compared to the other parameters. Thus similar grid parameters were used for all the IFD runs. The receptor TM helices 1–7 are coloured in VIBGYOR colour (Violet, Indigo, Blue, Green, Yellow, Orange and Red). Figure has been generated using PYMOL (The PyMOL Molecular Graphics System, Version 1.5.0.4 Schrödinger, LLC).

**Fig 2:**
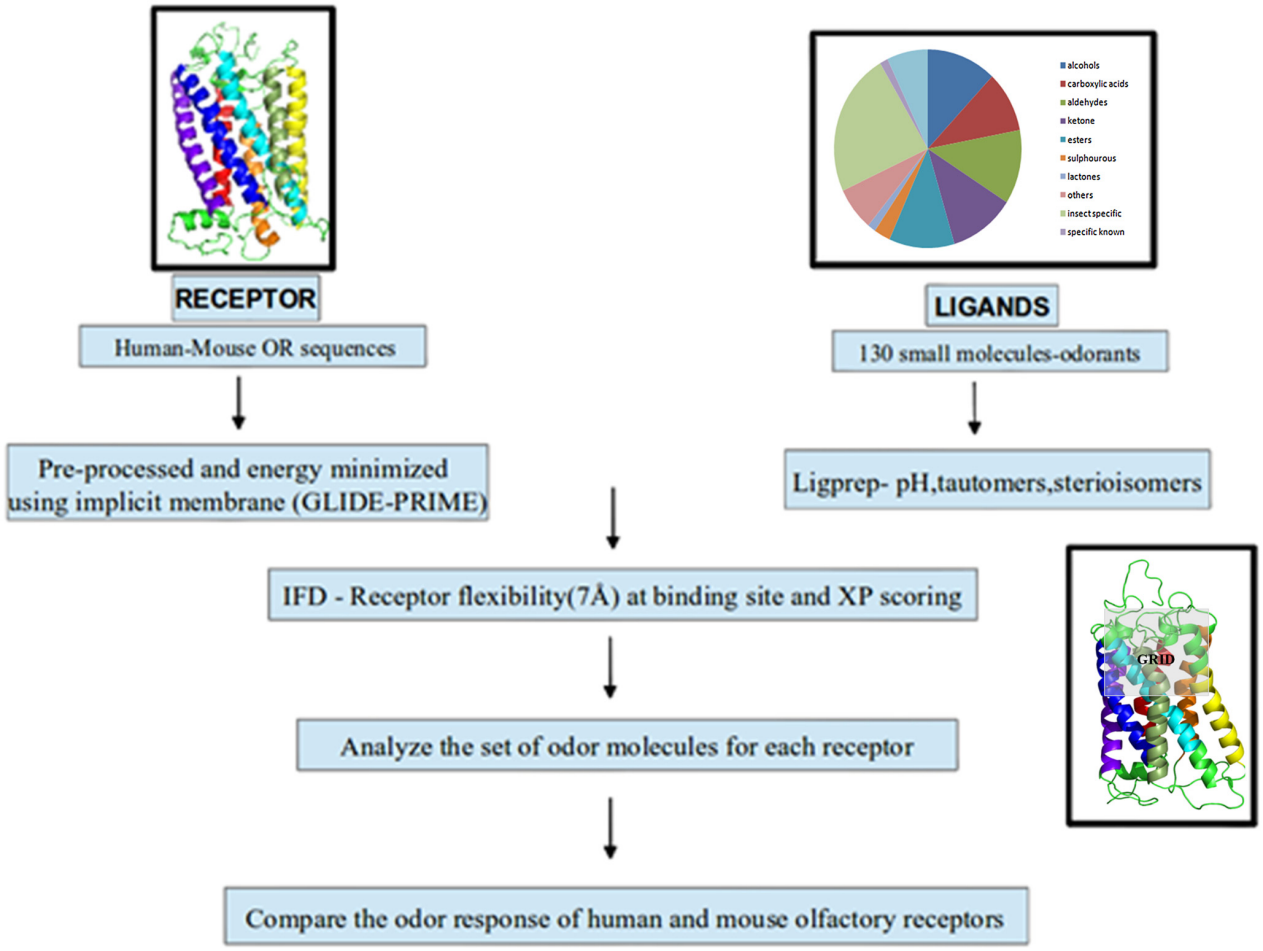
Induced Fit Docking Protocol. This figure represents the methodology followed for Induced Fit Docking. Ten pairs of human-mouse ORs were used as receptors and the 125 odorants as ligands and IFD was carried out using XP scoring. The odor profile for all the receptors obtained using IFD has been represented as heat map (Fig 8).

The induced-fit docking module of Schrodinger GLIDE software (**Schrödinger Release 2013–1**:, version 2.6, Schrödinger, LLC, New York, NY, 2013) was employed for docking 125 ligands to 10 pairs of closely related human and mouse olfactory receptors [23], [53]. The Schrödinger suite provides the opportunity to analyse GPCR-like membrane proteins in implicit and explicit membrane environments, thus mimicking the biological environment of these proteins. The homology models of ORs were energy-minimized in implicit membranes for further use in docking studies [39]. The docking protocol was standardized using the prior information on mouse eugenol receptors. The binding pocket of class A GPCRs are known from several studies [6], [54]. Table 2 shows the parameters that were employed to standardize the protocol of induced-fit docking for ORs using the mouse eugenol receptor and its ligand. The fifth parameter was chosen as the best, since it yields the best score for the known receptor-ligand complex. On the basis of this standardization, large-scale induced-fit docking was carried out using the grid made of all the residues in TM 3, 4, 5 and 7 in the upper half of the receptor in the membrane bi-layer which covers the known binding site of any given OR/GPCR protein (Fig 1). The side chains of residues, which are within 7Å of the initial ligand binding pocket, were given flexibility so as to induce conformational flexibility in the receptor. The residues in the receptor were scaled to 0.70 for Van der Waals interaction while for ligands it was scaled to 0.50 of the existing Van der Waals interaction scores. The XP scoring [31] of Schrödinger IFD module was used to score the final ligand-receptor complexes. The receptor-ligand pair with the highest score (gscore) was selected to compare the best binding mode for the selected receptor pairs. All the 125 receptor-ligand poses for a given receptor were ranked in the descending order of gscore. The ligand profile for each olfactory receptor was used for comparison across ORs and validation of the protocol (Fig 2).

**Table 2:** The different parameters used for standardization of IFD protocol. Parameter 5 (marked in bold) shows the highest score for binding of known agonist, eugenol to mOR-EG. Parameter 5 was thus chosen for further IFD analysis.

S.No.	GRID	Parameter	Gscore for Eugenol (kcal/mol)
**1**	Ser 113 as grid centre	No constraints	-5.96
**2**	Residues known to be at the binding site as grid centre (Ser 113, Phe 182, Phe 203, Phe 206, Asn 207, Leu 212, Phe 252, Ile 256 and Leu 259)	No constraints	-6.98
**3**	Ser 113 as grid centre	H-bond with Ser 113	-3.22
**4**	Residues known to be at the binding site as grid centre (Ser 113, Phe 182, Phe 203, Phe 206, Asn 207, Leu 212, Phe 252, Ile 256 and Leu 259)	H-bond with Ser 113	-7.09
**5**	**The upper half of the protein facing the extracellular mileu (in membrane) as grid**	**No constraints**	**-8.84**
**6**	Different rotameric states of Ser 113	No constraints	< -3.00

### Molecular Dynamics (MD) Simulations of Mouse OR-EG

The methodology for molecular dynamics simulations is as follows. The mouse OR73 models, in both ligand-bound and unbound form, were energy-minimized in an implicit membrane environment until convergence. The energy-minimized structures were then used as the start point for MD simulations. The MD simulations were carried out using DESMOND module of the GLIDE software [55] for 20 ns using the OPLS_2005 force field in the presence of 1-palmitoyl-2-oleoylphosphatidylcholine (POPC) lipid bilayer and standard NPT conditions. The protein was solvated in an orthorhombic box with periodic boundary conditions by adding TIP3P water molecules. The initial equilibration was carried out using default protocol of restrained minimization followed by molecular dynamics simulations for 20 ns.

## Results and Discussions

### Optimization of Docking Protocol

**Fig 3:**
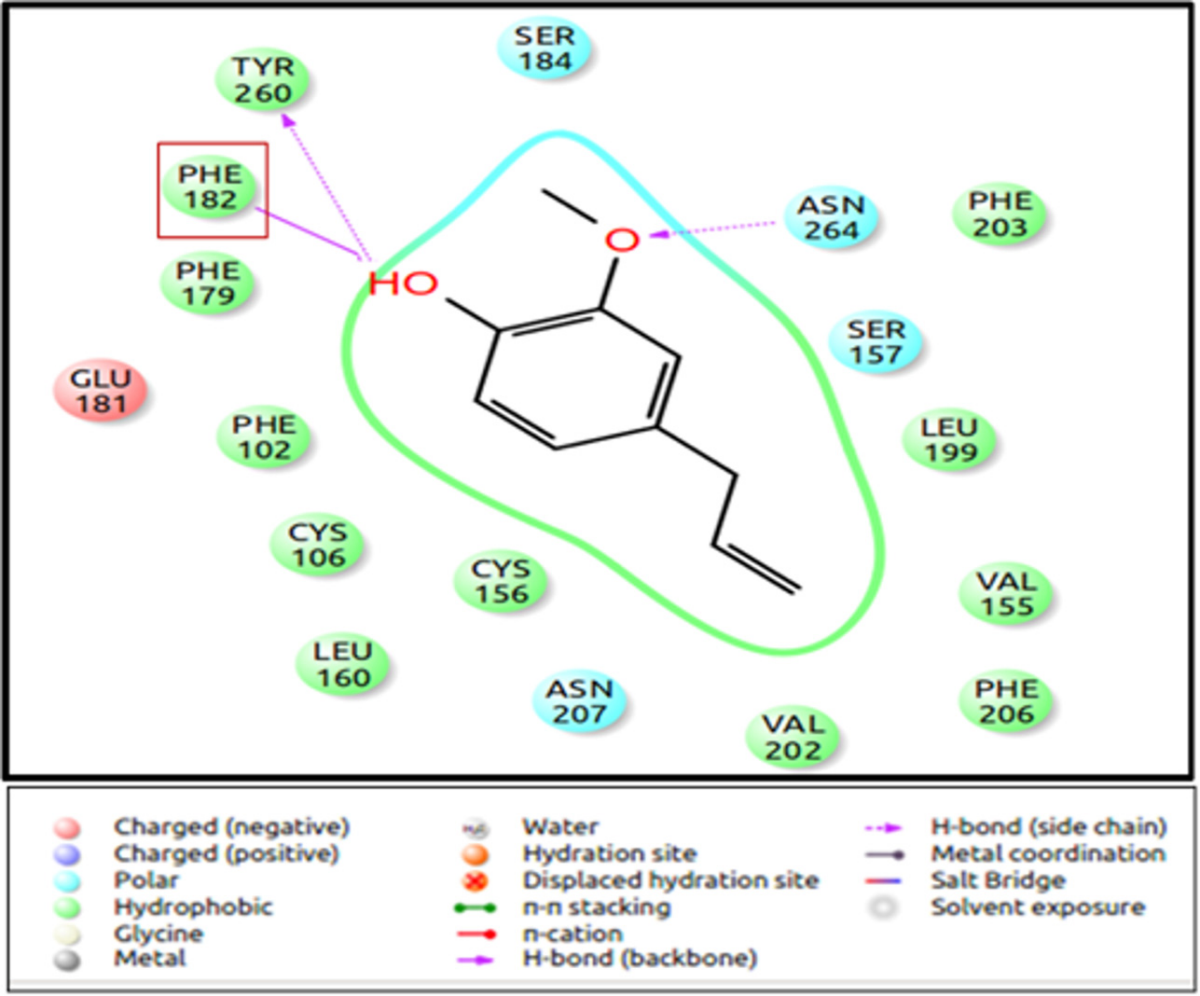
The binding mode of eugenol to mOR-EG. The figure shows the binding site of eugenol to mOR-EG. Phe 182 residue forms a H-bond with the—OH group. Other interacting residues are Tyr 260 and Asn 264, while other residues contribute to the hydrophobic pocket required for odorant binding. The figure is obtained using the “Ligand Interaction Diagram” of the GLIDE software (**Schrödinger Release 2013–1**:, version 2.6, Schrödinger, LLC, New York, NY, 2013).

Mouse eugenol receptor (MOR73/mOR-EG), isolated from olfactory receptor neuron was found to respond to eugenol, using calcium imaging studies, in heterologous cells, as well as *in vivo* studies [35]. The protein sequence of the receptor is available at NCBI (www.ncbi.nlm.nih.gov). Since it is a sufficiently well-characterised system, a homology model of the mOR-EG was built using the methodology described in our earlier analysis [39] [56] and used for evaluation of our docking protocol. The set of 125 ligands were docked to the mOR-EG receptor using the IFD protocol. The eugenol molecule is known to bind in the pocket made by TM 3, 4, 5 and 7 and interacts with mainly hydrophobic residues. Ser 113, Phe 182, Phe 203, Phe 206, Asn 207, Leu 212, Phe 252, Ile 256 and Leu 259 were observed at the binding pocket. Mutation of Ser 113 to Valine resulted in a loss of response to eugenol in one study and increase in EC_50_ value in another study [35] [37]. Further, the mutation of the residue Phe 182 resulted in complete loss of response of receptor to eugenol [37]. Different grid parameters were used (Table 2) to obtain a parameter which would yield highest score for binding of eugenol to the receptor and also include all the residues known to be involved in ligand binding in the binding pocket. In 90% of the poses, Ser 113 was not found to interact with the ligand, but was present at a distance of 6Å around the ligand. The receptor with different rotameric states of Ser 113 (S1 Fig) was used as the input for IFD, to check if change in rotameric state of the residue results in its interaction with ligands. However, changing the rotameric state of Ser 113 did not change its binding affinity to the ligand. Phe 182, however, was found to form H-bonds with more than 80% of the ligands including eugenol (Fig 3). All the other residues shown to be at the binding site from earlier studies were similar in our evaluation too. The parameter 5 gave the best binding energy for eugenol and was thus selected for further large scale docking analysis (Table 2).

### Olfactory Receptor ‘Orthologs’ with High Sequence Identity Do Not Share Similar Ligand Binding Profile

**Fig 4:**
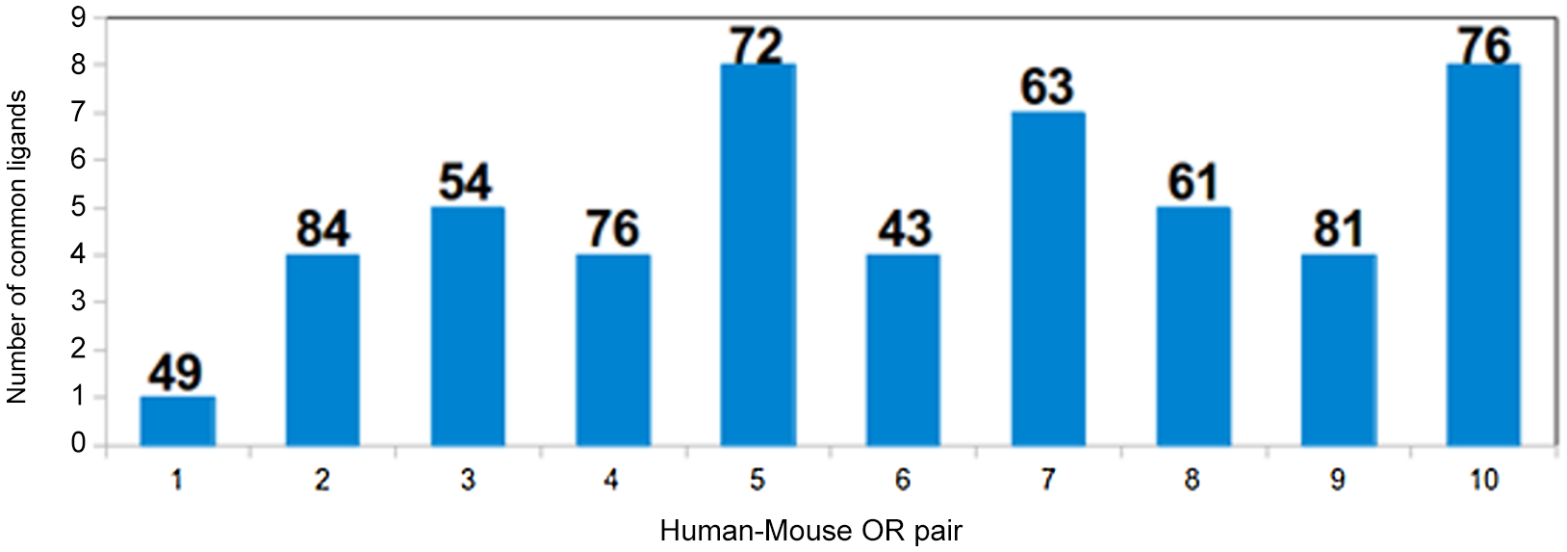
The number of common odorants among the best ten high scoring odorants for 10 human-mouse OR pairs. The figure shows the number of common ligands picked by ten OR pairs. The OR pair with highest sequence identity (Pair 2) has 4 common ligands while OR pair 5 and 10 have eight common ligands. The sequence identity of each pair is marked on top of the bar in the graph.

**Fig 5:**
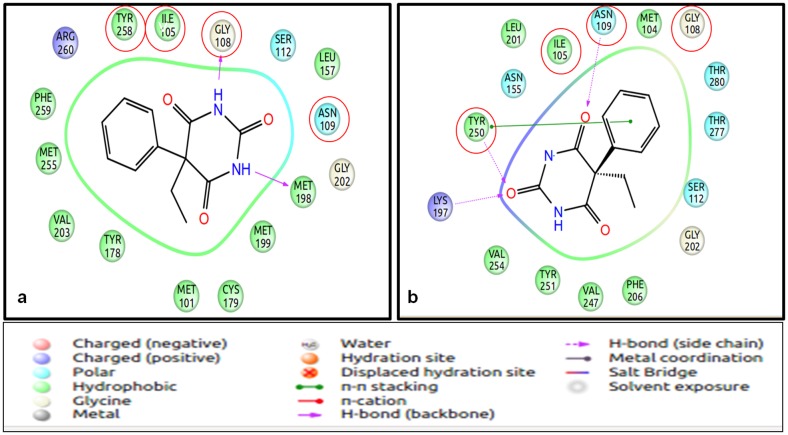
Odorant (Helional) binding residues of OR pair 2. (a): The odorant binding residues of human OR1A1. (b): The odorant binding residues of mouse OR18480066. The residues circled in red are the ones that are equivalent (identical) to both human and mouse ORs. The variability in the binding site results in differential responses and orientation of ligand binding of the two closely related ORs to the given odorant. The figure is obtained using the “Ligand Interaction Diagram” of the GLIDE software (**Schrödinger Release 2013–1**: version 2.6, Schrödinger, LLC, New York, NY, 2013) and PyMOL (The PyMOL Molecular Graphics System, Version 1.5.0.4 Schrödinger, LLC).

Ten pairs of closely related human-mouse olfactory receptors were selected for docking analysis. The receptor pairs had varying sequence identity. The highest identity between a receptor pair was 84%, while the lowest was 43% (Table 3). The true orthologs (Pairs 2, 4, 5 and 7) have been marked with ‘*’ (Table 3). This varying sequence identity in the dataset helped us analyse the possibility of whether highly similar OR sequences respond to similar ligands. The ligand-binding profiles for the first ten highest scoring ligands were compared for all OR pairs (Fig 4). It was observed that the OR pair with highest sequence identity (84%) has four common ligands out of ten best scoring ligands, while the OR pair with 72% and 76% sequence identity would respond to eight common ligands out of best ten scoring ligands. The ligand clusters were then analysed to check whether the ten high scoring ligands for the receptor pair with highest sequence identity belongs to the same cluster (Table 4), which wouldindicate that the response of receptors depends on chemical composition of the odorant and not on the odor emitted by the odorant. The receptors with highest sequence identity neither respond to common ligands nor to ligands belonging to similar clusters. This confirms that subtle changes at binding site compositions could result in differential odorant binding and odor detection. Such conclusions have been arrived at by several studies involving OR response to odorant under different circumstances. OR genetic polymorphism is known to alter function and, on an average, two individuals have functional differences at over 30%, suggesting that a given olfactory receptor with minor allelic variations across individuals of the same species could exhibit difference in responses to similar ligands [57]. Eighty seven percent of human-primate orthologs and 94% of mouse-rat orthologs showed differences in receptor potency to an individual ligand [7]. Despite high overall sequence identity (of 84%), only four residues are identical at the binding site of OR pair 2, while other residues are different. This difference in the local chemical environment could explain the varied response to a given set of odors of two closely related ORs (Fig 5). The electrostatic surface representation of the binding site of two receptors clearly shows the variation in the local chemical environment which could lead to different ligand binding profiles for the two receptors (S2 Fig). This difference in binding profiles may not be reflected by marked differences in gscores between human and mouse OR homologous pairs. The distribution of gscores (maximum, minimum and spread of gscores) for all the OR pairs has been represented as a boxplot (S3 Fig). The gscores range from -4 to -6 kcal/mol for the 10 pairs of ORs under study. The median values differ beween the human OR and its closely related mouse homologue by 1 unit; human_OR2 and mouse_OR2 (OR pair 2) with highest sequence identity (84%) retains identical median value.

**Table 3:** The receptor dataset used for the IFD study. The table shows the list of human and mouse ORs used for IFD analysis. Both the common name and GI ID of each OR is mentioned. The OR pair 2 has the maximum sequence identity of 84%. The orthologous pairs of ORs have been marked with an asterix '*'.

Human OR GI ID (common name)	Mouse OR GI ID (common name)	% Identity between the OR pairs	OR pair notation (based on the cluster number in phylogeny as mentioned in methodology)
289547623 (1A1)*	18480066 (MOR125-1)*	84	Pair 2
**53828670 (2Y1)**	18480552 (MOR256-24)	81	Pair 9
145279179 (8K5)*	18479442 (MOR187-1)*	76	Pair 4
**52317190 (2AK2)**	18480490 (MOR285-1)	76	Pair 10
52353290 (8H1)*	18479794 (MOR206-1)*	72	Pair 5
52546691 (11H1)*	18480158 (MOR106-5)*	63	Pair 7
**52353951 (0J3)**	18480958 (MOR267-13)	61	Pair 8
**52317182 (10S1)**	18480630 (MOR223-5)	54	Pair 3
**116642873 (52A1)**	18480856 (MOR24-3)	49	Pair 1
**153791572 (13A1)**	18479466 (MOR255-1)	43	Pair 6

**Table 4:** The first ten high scoring ligands for human-mouse OR pair 2. The human-mouse OR pair 2 has the maximum sequence identity of 84%. The highest scoring ligand is Helional for the two ORs, while there are only 4 common ligands out of the ten high scoring ligands (shown in bold font). The ligands that are dissimilar are from different clusters in ligand clustering analysis (Column 3 and 6).

Odorant	Gscore kcal/mol (Human OR GI: 289547623 (1A1)	Ligand Cluster Number	Odorant	Gscore kcal/mol (Mouse OR GI: 18480066 (MOR125-1)	Ligand Cluster Number
**Helional**	-8.51	15	**Helional**	-10.96	15
Ethyl-vanillin	-7.32	8	**Androstadienone**	-9.50	23
Hedione	-7.30	33_4	Androstenone	-8.36	23
Cumarin	-7.22	9	Nonanoic acid	-7.71	33_2
**Methyl salicylate**	-7.17	14	Indole	-7.56	12
**Piperonyl acetone**	-7.13	18	Methyl salicylate	-7.56	14
Thymol	-7.09	17	Piperonyl acetone	-7.35	18
Vanillin	-7.08	8	Butyric acid	-7.23	31
Menthol	-7.08	21	Lyral	-7.21	27
**Androstadienone**	-7.04	23	Octanoic acid	-7.18	33_2

### Ligand Dataset

**Fig 6:**
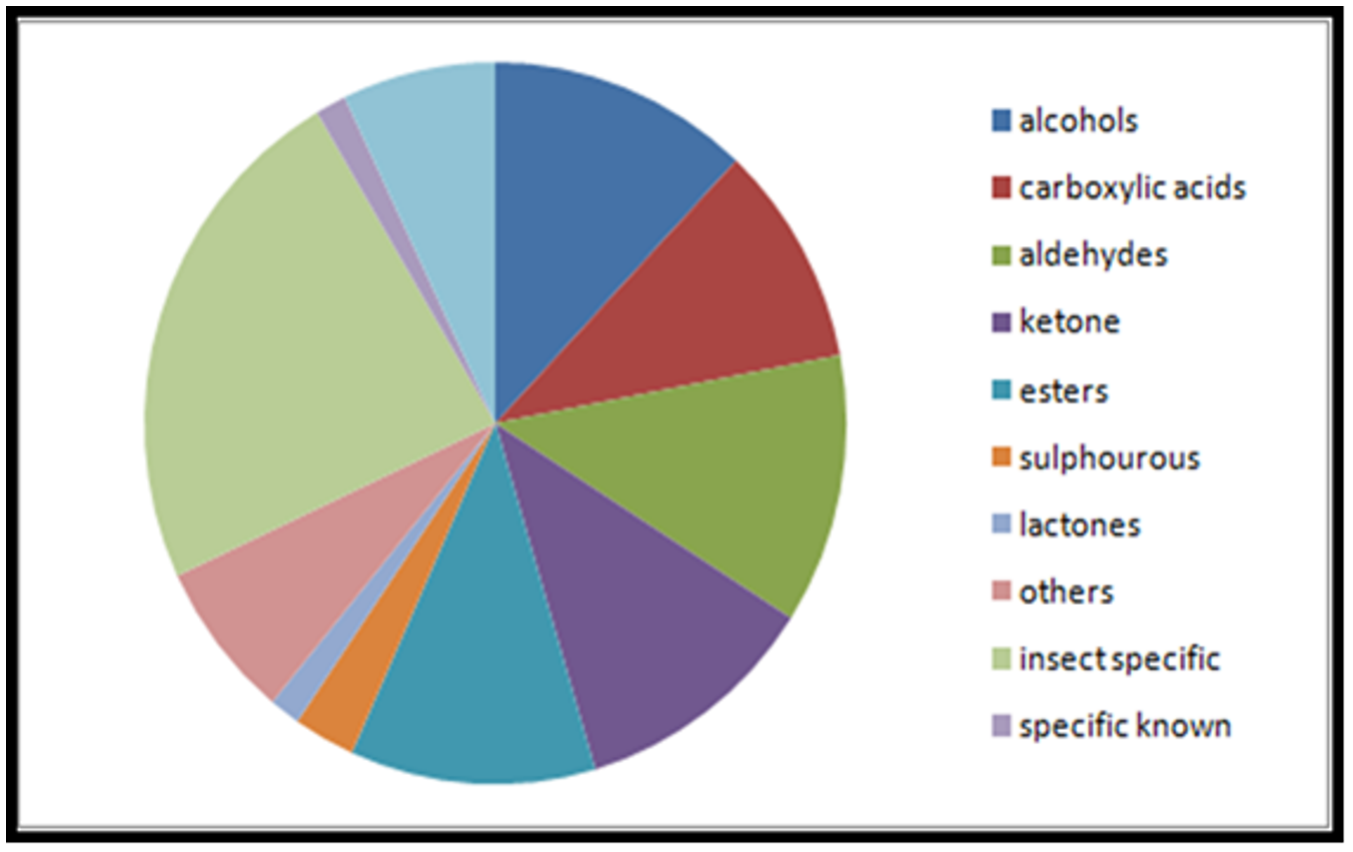
The distribution of odorants (125) into different chemical classes. The odorants belonged to different chemical classes with varying length of carbon chains. Few specific odorants that induce responses from insect ORs and mammalian ORs were grouped separately to understand their receptor binding activity.

**Fig 7:**
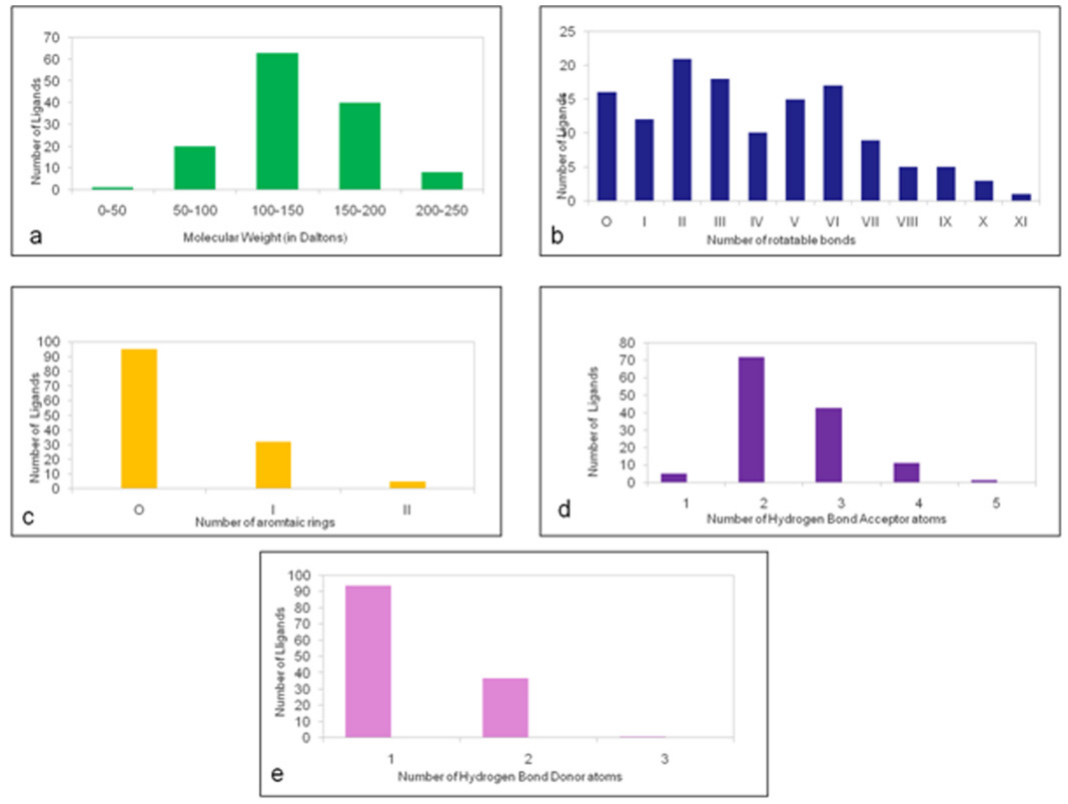
The distribution of MOLPRINT2D features of the odorants. (a) shows the range of molecular weight of the odorants. Most of the odorants have a molecular weight between 100–150 Daltons. (b) shows the number of rotatable bonds present in the given set of odorants. The number of rotatable bonds varies from 1 to 11. (c) shows the number of aromatic rings present in the odorants. 80% of the odorants are aliphatic. (d) and (e) show the number of hydrogen bond acceptors and donor atoms respectively in the odorants. There are a maximum of 5 hydrogen bond acceptors and 3 hydrogen bond donor atom in the odorants.

The ligand dataset consists of 125 odorant molecules belonging to various chemical classes like alcohols, ketones, carboxylic acids, aldehydes and sulphur containing compounds (Fig 6). The ligands were clustered using the canvas module of Schrödinger software into 36 unique clusters. The clustering was analysed at merging distance ranging from 0.1 to 1.0 at regular intervals of 0.5 (Table 5). At each of the merging distances, the clusters were manually checked to confirm that ligands with similar features were clustered into a group. The clustering that resulted in maximum number of similar ligands in a given cluster was selected for further analysis. The merging distance of 0.85 yielded 36 clusters and was used for further studies. The cluster 33 had 55 aliphatic odorant members in it and it was further divided into 11 sub-clusters based on the number of carbon atoms. The number of ligands in each cluster is given in Table 6. Based on MOLPRINT2D, the ligands were classified based on their molecular weight, number of rotatable bonds, number of aromatic rings and number of hydrogen bond donors and acceptors. More than 60 of the ligands have a molecular weight between 100–150 Daltons. The ligands contain 1–11 rotatable bonds while 75% of the dataset contains aliphatic chains. Seventy odorants contain at least two hydrogen bond acceptor groups, while 80 ligands contain at least one hydrogen bond donor group (Fig 7). The ligand clusters were further used to compare odor-binding profiles of OR proteins under study. Binding of similar odorants or odorants belonging to the same clusters to a given OR will indicate common binding modes.

**Table 5:** The different merging distances used for clustering of ligands. The ligand clusters obtained at a merging distance of 0.85 shows the presence of highly similar ligands in a given cluster and thus has been used for further analysis.

Merging Distance of Clustering	Number of Clusters
0.1	96
0.2	96
0.25	96
0.3	96
0.35	96
0.4	89
0.45	84
0.5	81
0.55	78
0.6	72
0.65	67
0.7	59
0.75	48
0.8	39
**0.85**	**36**
0.9	24
0.95	13
0.99	5

**Table 6:** The number of ligands in each of the 36 ligand clusters obtained by clustering (as mentioned in methods). Cluster 33 has 55 aliphatic odorant members in it and thus it is further subdivided into 11 subclusters based on functional groups of the odorants.

Cluster Number	Number of Ligands
**1**	1
**2**	1
**3**	1
**4**	1
**5**	2
**6**	3
**7**	4
**8**	4
**9**	1
**10**	2
**11**	1
**12**	3
**13**	1
**14**	4
**15**	1
**16**	1
**17**	2
**18**	3
**19**	2
**20**	9
**21**	2
**22**	4
**23**	3
**24**	1
**25**	1
**26**	1
**27**	3
**28**	1
**29**	1
**30**	5
**31**	3
**32**	9
**33**	55
**33_1**	2
**33_2**	7
**33_3**	12
**33_4**	1
**33_5**	3
**33_6**	6
**33_7**	9
**33_8**	1
**33_9**	1
**33_10**	8
**33_11**	5
**34**	1
**35**	1
**36**	1

### Induced Fit Docking

#### Induced fit docking of 10 homologous human-mouse OR pairs

**Fig 8:**
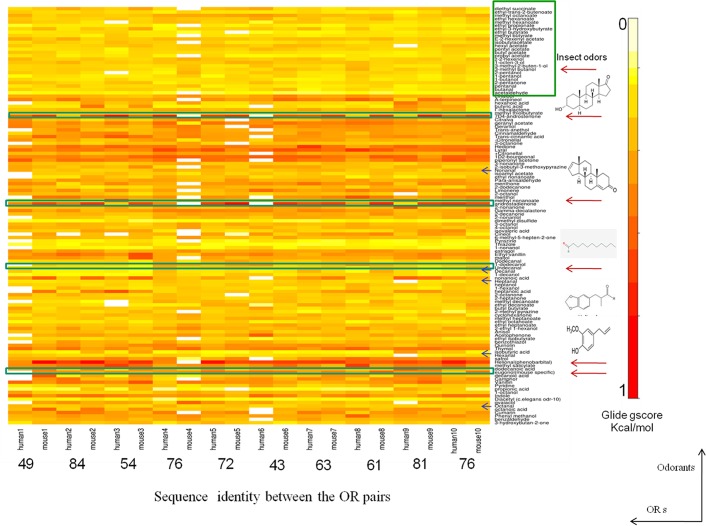
Heat map of the odorant profile of 10 human-mouse OR pairs. X-axis shows the human-mouse OR pair and the sequence identity between human-mouse OR pairs. Y-axis indicates the number of 130 odorants used in this study. The heat map is obtained using the gscore (kcal/mol) of interaction of each ligand to the given receptor. The scores have been normalized between 0 to 1 as shown in the scale. The odorants for which experimental data are available (Steroids, Helional, Undecanal, Eugenol and Citronellol) have been marked with a red arrow. Insect ORs have been marked in a green rectangular box. The heat map has been generated using R software.

One hundred and twenty five odorant molecules, as mentioned earlier, were docked to each of the twenty olfactory receptors individually using the IFD module of GLIDE Schrödinger software (**Schrödinger Release 2013–1**:, version 2.6, Schrödinger, LLC, New York, NY, 2013). Each IFD run takes upto six days on an I7 Linux machine with 2 processors. For each receptor, 125 or more complexes were generated based on the different tautomeric states of ligands. The table of energies has been reproduced as a **heat map** for visualization (Fig 8).

The average energy of OR binding to odorants is in the range of -4kcal/mol to -6kcal/mol. The average energy of interaction between human ORs and the odorants is -4.85kcal/mol, while for mouse ORs it is slightly higher, -5.09kcal/mol. The average difference in the binding of odorants between closely related human and mouse OR pairs was calculated. The pair with the highest sequence identity (Pair 2) has the minimum difference of average binding (Table 7) scores indicating overall similarity in binding mode between closely related OR sequences. The odorants with the highest scores for the 20 ORs belong to varied clusters of the ligand clustering data, perhaps since chemically similar odorants exhibit different odors and a given OR recognizes odorants based on shape or odor similarity.

**Table 7:** Average difference in binding energies (kcal/mol) of odorants to 10 human-mouse OR pairs. The average binding energies of 125 odorants to each of the ORs were calculated and the difference in the average energy between each human-mouse OR pair has been reported. The OR pair 2 (with the highest sequence identity of 84%) has the minimum difference in binding energy.

OR pair	Average difference in binding energies to odorants(kcal/mol)	% Identity between the OR pairs
**OR pair 1**	0.59	49
**OR pair 2**	0.01	84
**OR pair 3**	0.08	54
**OR pair 4**	1.97	76
**OR pair 5**	0.45	72
**OR pair 6**	1.74	43
**OR pair 7**	0.32	63
**OR pair 8**	1.17	61
**OR pair 9**	0.03	81
**OR pair 10**	0.65	76

#### Validation of docking protocol

In this section, we summarize the data on OR-odorant interactions known till date in the light of our computational study of OR-ligand modeling.

The **mOR-EG** receptor is known to respond to eugenol and compounds belonging to various chemical classes (vanillin-like compounds, polycyclic compounds and benzene derivatives etc.) [37]. The results using the IFD protocol mentioned above identifies similar vanillin-like, polycyclic and aromatic compounds (Helional, Ethyl-vanillin and Piperonyl acetone) to be high scoring ligands as compared to eugenol (Tables 8 and 9).

**Table 8:** The ten high scoring odorants for mOR-EG. Helional, Ethyl-vanillin and Eugenol are the experimentally proven ligands for mOR-EG which occur among the top five best scoring odorant-receptor interactions.

Odorant	Gscore (kcal/mol) of binding to mOR-EG
Helional	-11.87
Ethyl-vanillin	-9.74
Piperonyl acetone	-8.96
**Eugenol**	**-8.84**
Menthol	-8.49
Cumarin	-8.15
Decanoic acid	-8.08
Cineol	-8.07
Acetophenone	-7.52
2-Methyl pyrazine	-7.18
2-Octanone	-6.08

**Table 9:** OR-odorant interactions reported till date. The OR-odorant interactions reported in studies done so far has been mentioned in this table. The remarks column indicates the results from the current study that correspond to the known data on OR-odorant interactions.

OR name	Ligands	Remarks	Reference
mOR-EG (Mouse)	**Eugenol**,methyl isoeugenol,lyral. **Undecanal** (antagonist), 2-methoxy-4-ethylphenol;, 2-methoxy-4-methylphenol;, eugenol acetate,eugenol ethyl ether, **ethyl vanillin**, Mousse Cristal, 4-hydroxy-3-methyl benzaldehyde	mOR-EG**-Eugenol** complex is in the top 5 best binding receptor-ligand complexes in the current study. **Undecanal** is found to bind better than many selected odorants and in the same binding pocket as the known ligands suggesting competitive inhibition. **Eugenol** and compounds structurally similar to it such as **ethyl vanillin** have been ranked in the top 10 best binding odorants to mOR-EG. (Please refer Table 8).	[8], [15] and [35].
MOR-EV (Mouse)	Ethyl-vanillin	-	Same as above
MOR-23 (Mouse)	Lyral	-	Same as above
MOR1-1,MOR105-1,MOR106-1	(+)-2-Phenylbutyric acid, (+)-Camphor, (+)-Carvone, (+)-Dihydrocarvone, (+)-Fenchone, (-)-2-Phenylbutyric acid, (-)-Camphor, (-)-Carvone (-)-Fenchone, **(-)-b-Citronellol**, 1-Decanol, 1-Heptanol, 1-Hexanol, 1-Nonanol, 1-Octanol, 1-Pentanol, 2-Coumaranone, 2-Heptanone, 2-Hexanone, 2-Nonanone, 2-Octanone, 2-Pentanone, 23-Hexanedione, 3-Heptanone, 3-Octanone, 34-Hexanedione, 4-Chromanone, 4-Hydroxycoumarin, Acetophenone, Allyl benzene, Allyl heptanoate, Allyl phenylacetate,Amyl hexanoate, Benzene Benzophenone, Benzyl acetate, Butyl butyryllactate, Butyl formate, Coumarin, Cyclohexanone, Decanal, Decanoic acid, Dihydrojasmone, Ethyl isobutyrate, Geraniol, Heptanal, Heptanoic acid, Hexanal, Hexanoic acid, Hexyl acetate, Lyral, Nonanal, Nonanethiol, Nonanoic acid, Octanal, Octanethiol, Octanoic acid, Pentanoic acid, Phenyl acetate, Prenyl acetate, Propionic acid, Vanillic acid, g-Caprolactone	The interaction between **OR1A1** and the **odorant (-) Citronellol** is found to score better than the interaction between OR1A1 and (+) Citronellol in the current study. The review reports (-) Citronellol to be an agonist for OR1A1 while the other stereoisomer do not activate the OR (please see text for details).	[8].
MOR107-1,MOR128-2,MOR129-1			
MOR136-1,MOR139-1,MOR140-1			
MOR15-1,MOR161-1,MOR162-1			
MOR170-1,MOR18-1,MOR180-1			
MOR182-1,MOR184-1,MOR185-1			
MOR189-1,MOR2-1,MOR203-1			
MOR204-6,MOR205-1,MOR207-1			
MOR222-1,MOR223-1,MOR23-1			
MOR236-1,MOR25-1,MOR250-1			
MOR251-1,MOR253-1,MOR256-17			
MOR258-1,MOR259-1,MOR260-1			
MOR261-1,MOR268-1,MOR269-1			
MOR271-1,MOR272-1,MOR273-1			
MOR277-1,MOR30-1,MOR31-1			
MOR33-1,MOR37-1,MOR4-1			
MOR40-1,MOR41-1,MOR5-1			
MOR9-1,OR10J5,**OR1A1**,OR2C1,OR2J2,			
OR2M7,OR2W1,OR51E1,OR51E2			
,OR51L1,OR5P3 (53 Mouse ORs and 10Human ORs).			
M71 OR (Mouse)	acetophenone and benzaldehyde	-	[69]
S6 (Human)	Nonanedioic acid, Octanoic acid	-	[70]
S86 (Human)	Nonanoic acid, Octanoic acid, Heptanoic acid	-	Same as above
r-I7 (Rat)	Octanal, Heptanal	-	Same as above
m-I7 (Mouse)	Octanal, Heptanal	-	Same as above
OR17-40 (Human)	**Helional**	OR1D2 in the current study is also labeled as OR17-40 in certain studies. **Helional**-OR1D2 complex is in the top 10 of the best scoring receptor-ligand complex among the 125 other odorants used in the study.	Same as above
mOR912-93 (Mouse)	2-Heptanone	-	Same as above
mI-C6 (Mouse)	(−)Citronellal, β-Citronellol	-	Same as above
Olfr43 (Human)	(−)Citronellal, β-Citronellol	-	Same as above
**OR1G1 (Human)**	Nonanal,9-Decen-1-ol,1-Nonanal,camphor,n-butanal, 3-methylbut-1-yl ethanoate (isoamyl acetate), 3-methylbut-1-yl ethanoate (isoamyl acetate), 2-ethyl-1-hexanol, 1-nonanol,ethyl isobutyrate,γ-decalactone,nonanal,trans-anethol, piperonyl acetone, lyral and hedione, pyrazines, thiazols,cyclohexanone, Octopamine, isoamyl acetate, ethyl isobutyrate, tridecanal, 2-undecanone.	-	[38], [41], [65] and [66].
OR52D1 (Human)	methyl octanoate,ethyl heptanoate, 1-nonanol,2-nonanol,3-nonanone,3-octanone	-	Same as above
MOR42-3 (Mouse)	undecanal and nonanoic acid	-	[71]
	tetramethyl-hexadec-1-en-3-ol, 1-methyl-ethyl-2-phenylethanoate,ethyl-3-methyl-3-phenyl-oxirane-2-carboylate, 5-methyl-2-phenyl-hex-2-enal, 4-(4-hydroxy-4-methylpentyl)-cyclohexen-1-carbaldehyde		
Human ORs	**Aldehydes and Helional**	In this review psychometric function test was used to show that **Helional** is the most potent **Aldehyde** at a low odorant concentration. In the current study we find that of all the aldehyde group of odorants, Helional-receptor complexes have the highest average gscore (please refer Table 9).	[62]
OR7D4 and OR1D2 (Human)	**Bourgeonal, androstenone**,androstadionone.	The human OR7D4 and OR1D2 and known to be evolutionarily related and found to be expressed ectopically in testis. **Bourgeonal** is known to be the endogenous ligand for these receptors, while they do respond to **androstenone** and other testicular odorants. In the current study androstenone is reported to be the highest scoring OR-odorant complex for 1D2. Bourgeonal is found to bind in the same binding pocket as androstenone but with a lower binding score.	[60]
**OR1D2** (Human)	**Bourgeonal and Undecanal**	**Bourgeonal** is the known agonist for OR1D2 while **undecanal** is the antagonist. In the current study both bourgeonal and undecanal bind in the same binding pocket of the OR1D2. The OR-undecanal complex scores higher than the OR-bourgeonal complex indicating that undecanal may act as a competitive inhibitor.	[59]

**Fig 9:**
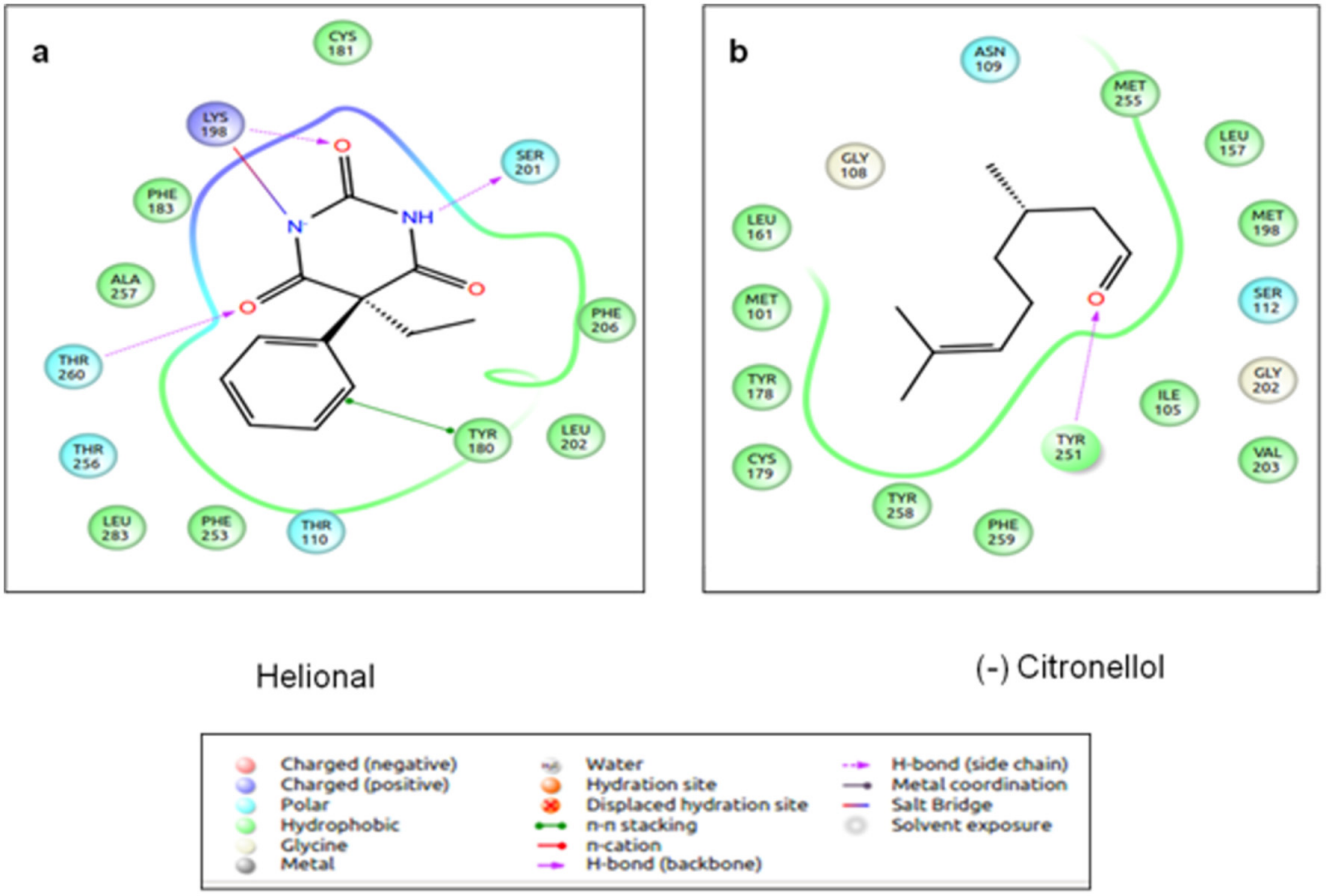
Binding mode of Helional and (-) Citronellol to human OR1A1. Helional (a) forms three hydrogen bonds and one salt bridge with the residues of OR1A1, while Citronellol (b) forms only one H-bond with the residues of OR. Helional is known to be the most potent alcohol for human ORs. The figure is obtained using the “Ligand Interaction Diagram” of the GLIDE software (**Schrödinger Release 2013–1**:, version 2.6, Schrödinger, LLC, New York, NY, 2013).

Human **OR1A1** (belonging to OR pair 2) responds to citronellol and helional even at lower concentrations when compared to aldehydes with 6–9 carbons atoms (Table 9) [58]. Among the different stereoisomers of citronellol, the receptor is more responsive to (-) citronellol than (+) citronellol. The hydrophobic binding pocket is very similar to the one observed in mOR-EG receptor. The TM 3, 4, 5, 6, and 7 are involved in interactions with the ligand. Gly 108, Asn 109 and Ser 112 are involved in interactions with the ligand and mutation of these residues results in a reduction of response to these odorants. These residues are found in the binding pocket of OR1A1, derived from the current OR-ligand docking protocol. Helional is the highest scoring ligand (-8.51kcal/mol) in this mini-virtual screening exercise, while (-) citronellol obtains a GLIDE score of -5.76kcal/mol, though the binding pockets for both ligands are similar in our analysis (Fig 9). (+) citronellol scores lower than the two above mentioned odorants. Comparing the residues at the binding pocket for the close homologue of OR1A1 in mouse (Fig 5), we observe that the four residues known to be important in ligand binding are common, while the rest of the binding pockets differ in the composition of residues. This variability at the functional site allows the closely related OR sequences to bind to myriad odorants.

**Fig 10:**
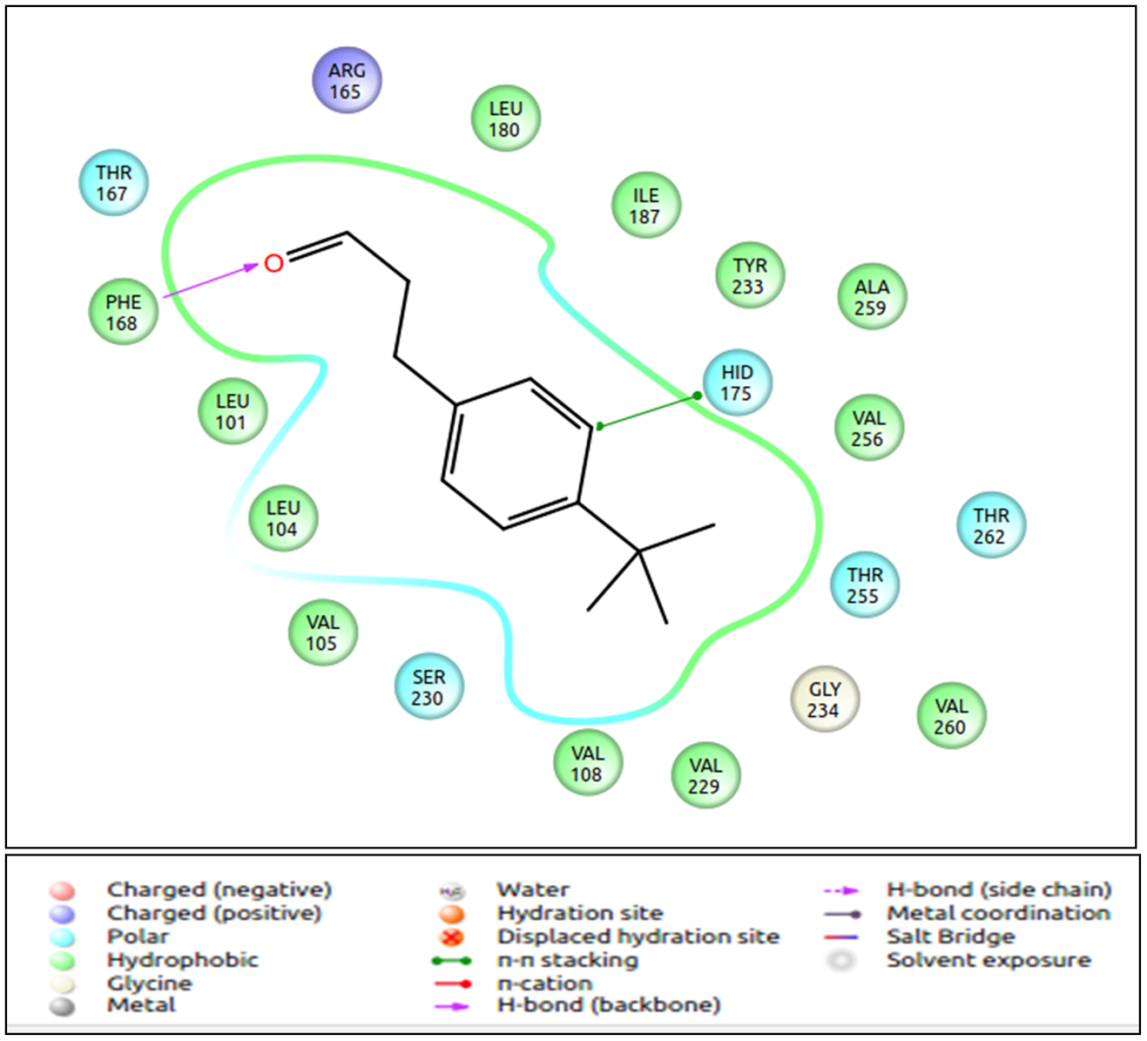
Binding mode of bourgeonal to human OR1D2. Bourgeonal is known to be the most potent ligand of human OR1D2. It forms a H-bond with the residue Phe168 of the receptor. It binds to the common GPCR binding pocket formed by TM3, 4, 5 and 7. The figure is obtained using the “Ligand Interaction Diagram” of the GLIDE software (**Schrödinger Release 2013–1**:, version 2.6, Schrödinger, LLC, New York, NY, 2013).

Human **OR1D2** is a receptor found in human spermatozoa [59]. It is known to respond to bourgeonal and is suppressed by undecanal (Table 9). The OR1D2 receptor is evolutionarily related to the human receptor 7D4, that detects steroids such as androstenone and androstadienone. Point mutations in OR7D4 result in variations in response to the known odorants across different individuals [60]. It is reported that OR1D2 also responds to steroid hormones with lesser efficacy as compared to OR7D4 (Table 9). In the docking analysis, androstenone and androstadienone are observed as the best scoring ligands for 1D2, with a GLIDE score in the range of -10kcal/mol, while bourgeonal binds with a score of -4.48kcal/mol. The binding pockets remain similar for both the odorants. This study confirms the fact that by subtle changes at the receptor binding site, the receptor can accommodate similar ligands. The difference in ligand binding scores could be because of the difference in the functional groups of the odorants (Fig 10).

**Fig 11:**
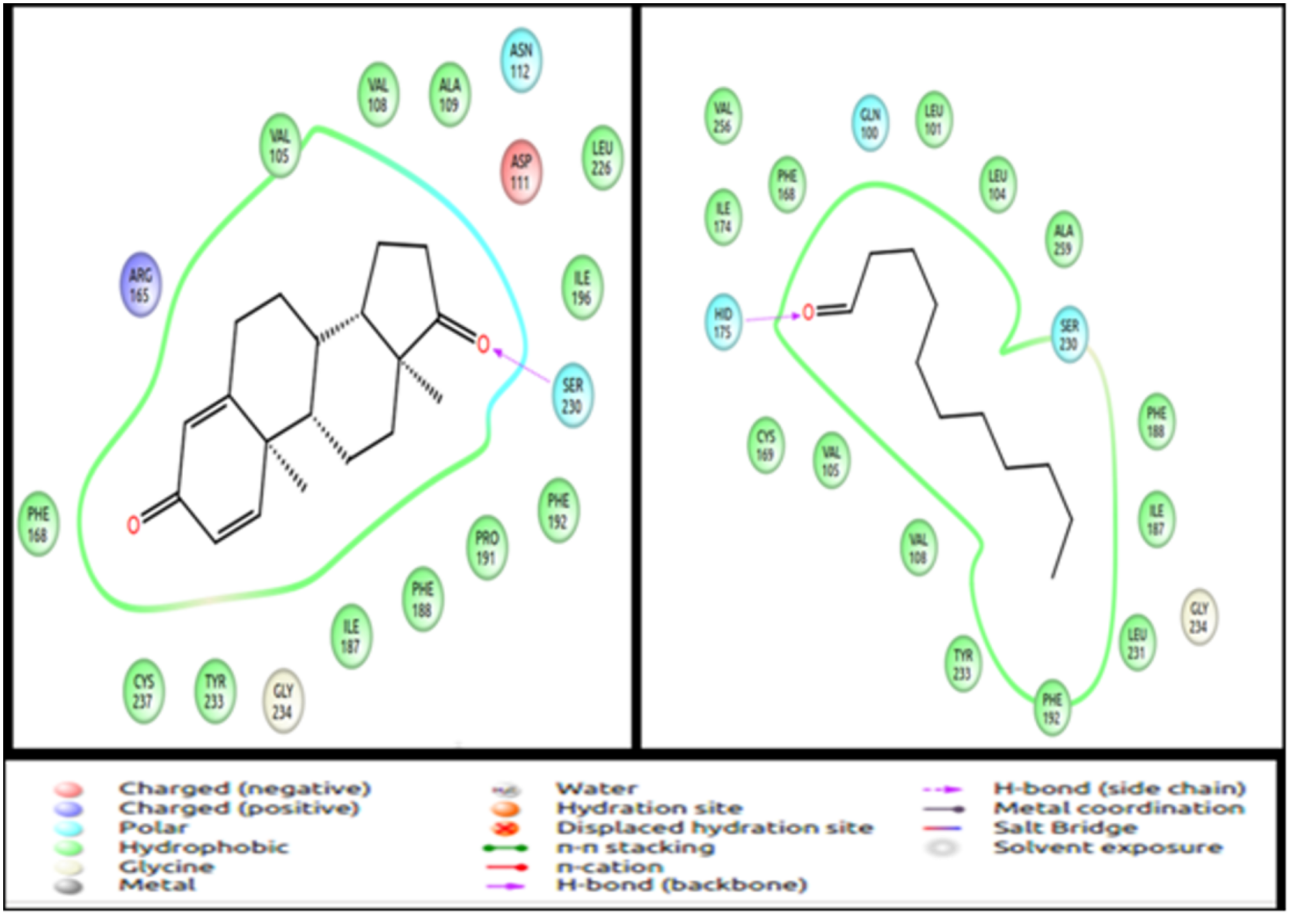
Binding mode of androstenone (agonist) and undecanal (antagonist) to the human olfactory receptor 1D2. Both the odorants bind in the same binding pocket but interact with different residues. Val108, Val 109, Phe 168, Ile 187, Ser 230, Tyr 233 and Gly 234 are the common residues at the binding site. The figure is obtained using the “Ligand Interaction Diagram” of the GLIDE software (**Schrödinger Release 2013–1**:, version 2.6, Schrödinger, LLC, New York, NY, 2013).

**Undecanal** is a known inhibitor for human olfactory receptor hOr17-4 (1D2) (Table 9) [59] [61] and for its homologue in rat, the I7 receptor. The human and the rat ORs are known to respond to bourgeonal, the agonist [15]. The gscore of undecanal interaction with the receptor 1D2 in this study is -5.2 kcal/mol, which is lower than binding affinity of the highest scoring pair (-10.73kcal/mol), but better than bourgeonal (-4.48Kcal/mol) which is the known agonist (inhibited by undecanal). The binding site of undecanal is same as the other high scoring ligands, suggesting competitive inhibition of these receptors by undecanal (Fig 11).

**Fig 12:**
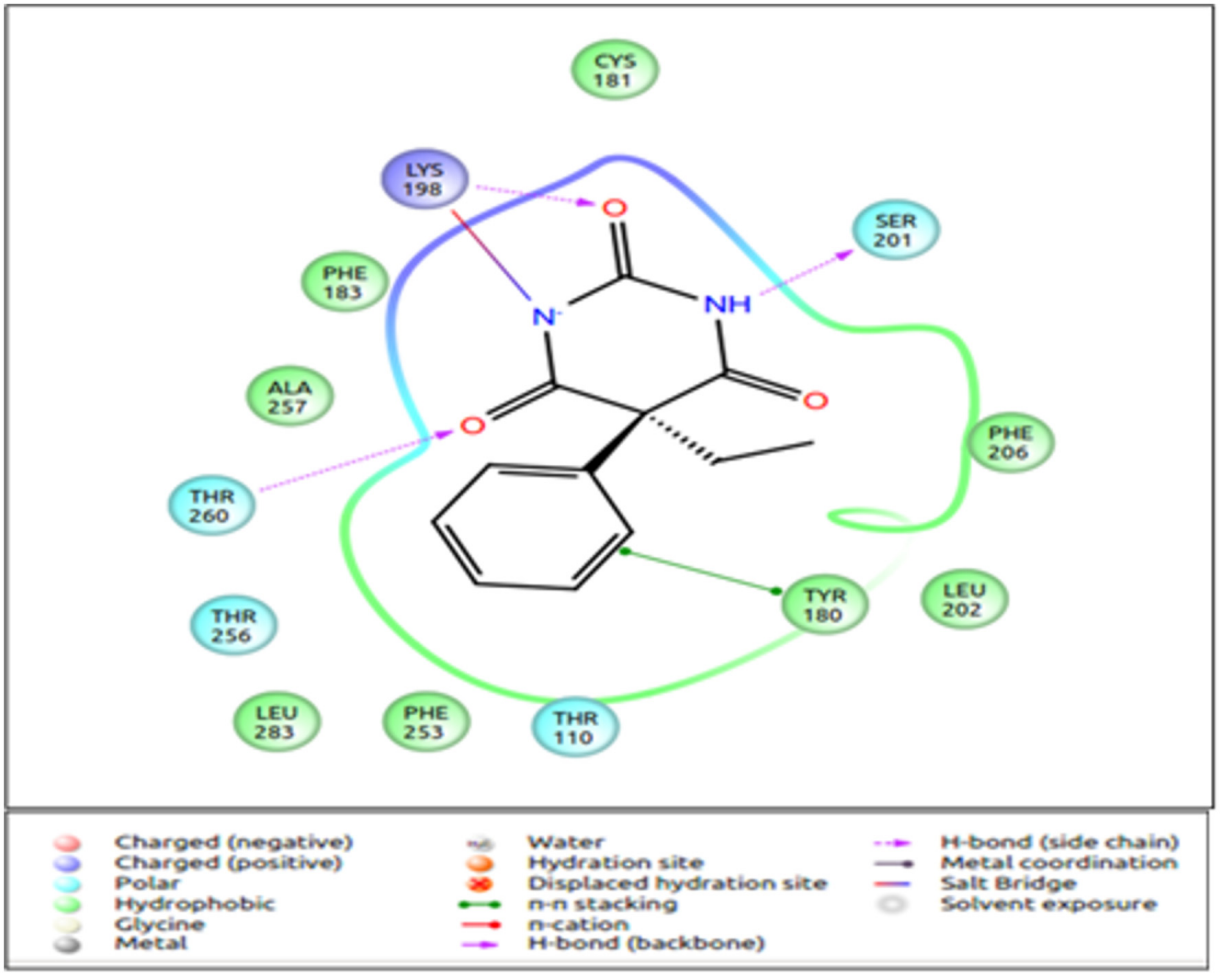
Best binding mode of Helional to OR. This interaction has the highest score in the IFD runs. There are three H-bonds and one salt bridge interaction between the odorant and the residues of the olfactory receptor. The figure is obtained using the “Ligand Interaction Diagram” of the GLIDE software (**Schrödinger Release 2013–1**:, version 2.6, Schrödinger, LLC, New York, NY, 2013).

**Aldehydes** of varying carbon length show high response by human olfactory receptors (Table 9) [62]. Helional is the most potent aldehyde when compared to butanal, hexanal, heptanal, octanal, nonanal and decanal. Helional has the highest average docking score (-5.66kcal/mol) for the twenty olfactory receptors under study by IFD. The average score for other aldehydes are as reported in Table 10. The highest score of helional is -12.26kcal/mol and it forms three H-bonds, one salt bridge and one pi-pi interaction (Fig 12), which results in the most stable interaction as compared to other ligands. Invariably, larger ligands would score better than eugenol due to higher extents of hydrophobic interactions.

**Table 10:** Average gscore (kcal/mol) for interactions between aldehydes in the odorant dataset to the 10 human-mouse OR pairs. The average binding energy of each of the aldehyde to the 20 ORs was calculated. Helional is known to be the most potent aldehyde as compared to aldehydes with 5–10 carbon atoms.

Odorant (aldehyde)	Average gscore (kcal/mol) of binding to 10 human-mouse OR pairs
**Helional**	**-5.66**
**Butanal**	-2.36
**Pentanal**	-2.21
**Hexanal**	-2.17
**Heptanal**	-2.35
**Octanal**	-2.75
**Nonanal**	-3.09
**Decanal**	-2.49

**Insect olfactory receptors** are known to detect odorants with lesser numbers of carbon atoms (2–5 carbon atoms), while mammalian odor detect odorants with higher numbers of carbon atoms (5–12 carbon atoms) [42] [63]. The ligand dataset consisted of 25 insect OR specific odorants, comprising about one-fifth of the whole dataset of odorants. The average energy score of insect odorants upon docking to the 10 human-mouse pairs is -4.15 kcal/mol, as compared to mammalian specific odorants which have an average energy score of -5.18 kcal/mol. This confirms that mammalian specific odors form better interactions to mammalian ORs and thus have a high binding score. Similar studies on insect ORs require the availability of homology models for many insect ORs and their co-receptors. Due to the inverted topology and varying loop lengths, it is difficult to obtain high quality homology models for several insect ORs [64]. Such a study will, however, be very important in understanding how insect vectors detect human hosts using the sense of smell.

### Antagonistic Activity of Odorants

Olfactory receptors exhibit a combinatorial code of response [1]. Odorant response varies when presented as single odorant and as a mixture of odorants. In a mixture, some odorants are known to antagonize the effect of other odorants and the response is the cumulative effect of all the odorants in the mixture. The antagonistic effect also depends on the neuron in which the OR is expressed. There has been no study to differentiate the perception of agonists and antagonists [15] as antagonists also bind to the ORs unlike the non-binders (which can be differentiated using the free energy of binding). The odorant which acts as an agonist for one OR could behave as an antagonist for another OR [41]. The response to antagonists may not necessarily lead to an inactive state of the receptor. It may result in a decreased response of the agonist and thus cannot be differentiated at the receptor expression levels. Antagonists tend to be structurally related to agonists. For example undecanal (an antagonist) is structurally similar to bourgeonal (an agonist) [41]. In nature, odorants exist as a mixture and very rarely as a single compound. Thus, in the docking studies to understand one to one OR-odorant relation, it becomes difficult to differentiate the antagonists from the agonist until one studies the activation of ORs using these ligands [65, 66]. In this study, it is observed that undecanal, a proven antagonist for the human OR1D2, scores higher than the endogenous ligand, bourgeonal. It is known to inhibit the response of OR1D2 to bourgeonal by binding to the same ligand binding pocket as that of bourgeonal [41].

### MD Simulations of Mouse OR-EG

MD simulations of the mouse OR-EG was performed as mentioned in methods. The energy drift of the ligand-bound form in the initial 10 ns is ~ -300kcal/mol while in the last 5 ns it is ~ -100kcal/mol. This suggests that the receptor in the ligand-bound form remains in a stable state throughout the simulation, without huge differences in the energy of the system. Overall, the ligand-bound form has lower energy throughout the simulations as compared to the unbound form (S4 Fig). We find the ligand-binding pocket is made up mostly of hydrophobic residues and few polar residues that form H-bonds. Ser 113, which is shown to be important in ligand binding [35, 37], is found to form a H-bond in about 35% of the overall simulation time (S5 and S6 Figs). We find that the residues at the binding site are spatially clustered and remain so throughout the MD simulation indicating that the ligand is bound firmly in a particular binding pocket and does not switch positions (S7 Fig).

### Database of Olfactory Receptors-Access to Receptor-Ligand Complexes

The Database of Olfactory Receptors (DOR database) (http://caps.ncbs.res.in/DOR) [67] contains information on sequences, phylogenetic analysis and homology models of olfactory receptors from five eukaryotic organisms. Models of the Receptor-Ligand complexes for the 20 olfactory receptors with each of the 125 ligands have now been included in the DOR database. The files are available in the ‘LIGAND DOCKING’ tab of the database. The user can download a compressed ‘tar file’ for each olfactory receptor and its ligand complexes. The olfactory receptors are labelled as per their ‘GI Ids’ (Table 3). The receptor-ligand complexes are in the PDB format and they are labelled based on the Pubchem code for each of the ligand used in the study (Table 1). The availability of all the protein-ligand complexes in the public domain will be helpful for a wide range of analysis on these classes of proteins.

## Conclusion

Previously, we had exploited distant relationships between ORs and GPCRs to arrive at three-dimensional models of 100 ORs using tools like homology modelling [39]. Olfactory receptors are known to have a combinatorial response to odors and OR-ligand discrimination has been recorded in literature only for a few ORs through careful experiments. In this paper, we selected 20 ORs of both human and mouse origin, and used docking and virtual screening of 125 known ligands to arrive at OR-ligand profiles. To the best of our knowledge, this is the first longitudinal large-scale computational study using docking to arrive at OR-ligand profile. Further, docking scores that correlate well with OR-ligand affinities known from experiments have been obtained. Eugenol and eugenol-like ligands were recognised as top-ranking ones by the current docking protocol. We have shown the selective non-affinity of *Drosophila* OR-ligands by mammalian ORs. The current docking protocol and scores are sensitive even to identify better ligands between stereoisomers like (+) and (–) citronellol. Known ligands and inhibitors could be correctly identified for MOR73, human OR1A1 and 1D2 using docking scores. We are currently predicting a large number of OR-ligand pairs whose relative affinities are yet to be tested.

Using a well-validated protocol, methods have been standardized to obtain an odorant profile, through mini-virtual screening, for a given olfactory receptor protein for a limited number of odorants. Olfactory receptors bind to myriad of odors and it is difficult to decode this complex combinatorial response process. Many OR-odorant profiles still remain undeciphered. *In silico* tools like homology modeling and induced fit docking provide us the advantage of inducing flexibility to both receptor and ligand. This creates a scenario very similar to the one that occurs biologically in a cell, wherein a receptor undergoes conformational changes to accommodate a given ligand. OR sequences exhibit great diversity. Homologous OR sequences do not respond similarly to a given set of odorants. A small change in residue composition at the binding site results in different odor profiles, which cannot be realised from the overall sequence identity of two ORs under question. The binding site and binding mode vary greatly across ORs. This helps the ORs recognize numerous odors in the environment. Another method that could be pursued is to introduce flexibility to the ORs using Molecular Dynamics (MD) simulations. The different conformations obtained for a receptor can then be used to identify a set of odorants that would bind to the receptor above a given energy threshold. Molecular Dynamics simulation study for a large data set of receptors (400–1000 mammalian ORs) is very computer intensive and time consuming. In this regard MD simulations were carried out for mouse OR-EG in the eugenol-bound and unbound form. We find that key interactions between this ligand and OR remain the same throughout 20 ns simulations.

ORs are known to be expressed in tissues other than oro-nasal cavity i.e. testis, lungs and pancreas [68]. Obtaining the odorant profile for such ORs will help us in understanding their role in the given tissue. ORs are known to be over-expressed in certain types of cancer and diabetes and it will be interesting to decode the function of such ORs that could then be used in pharmacological studies. From the current study, known ligands are observed to bind with a energy threshold greater than -4.5kcal/mol. This can be used as a cut-off to obtain ligand profile for a given odorant. Highly related OR pairs show *least* difference in average binding energy to the given set of odorants. This can be used to compare the odorant profiles of similar ORs, especially in cases where one of the OR has been de-orphaned. Induced fit docking protocol can thus be systematically used to understand the structural and functional divergence of olfactory receptor class of proteins.

## Supporting Information

S1 Fig The different rotameric states of Ser 113 residue of mOR-EG. Different rotameric states of Ser 113 were obtained using PRIME module of GLIDE. Ser 113 was shown to be important for binding of eugenol to mOR-EG. Different rotameric states of Ser 113 were used to check if the odorants form any bonded interaction with Ser 113 in its different rotameric states. The change in rotameric state of Ser 113 residue did not increase its proximity to the ligand. The figure is obtained using PyMOL (The PyMOL Molecular Graphics System, Version 1.5.0.4 Schrödinger, LLC).
(TIF)

S2 Fig Electrostatic surface representation of Mouse OR (18480066) and Human OR (1A1). Electrostatics is represented by calculated charge from red (acidic residues; -5 kbT/ec) to blue (basic residues; +5 kbT/ec) as in Adaptive Poisson—Boltzmann Solver (APBS) program in PyMOL (The PyMOL Molecular Graphics System, Version 1.2r3pre, Schrödinger, LLC.). The surface electrostatics at the binding site of human and mouse ORs (Sequence identity is 84%) is different and this explains the difference in varied ligand profiles of these receptors.
(TIFF)

S3 Fig Distribution of gscores for 20 Olfactory receptor proteins and their interaction with the 125 odor molecules. The distribution of gscores for OR proteins and the odor molecules has been represented as a Box-Whisker plot (prepared using R-scripts). The plot represents the spread of gscores for 125 odor molecules against each of the 20 OR proteins. The gscores range from -4 to -6 kcal/mol for all the ORs under study. For the OR pair (Pair 2) with highest sequence identity (84%) the median value of gscore is equal. The circles outside the plot represent the outliers. The OR proteins are numbered based on the pair they belong to. For eg: Human 1 and Mouse 1 belong to OR pair 1 under study.
(TIFF)

S4 Fig Graph representing the energy of the mouse OR-EG model for the 20 ns MD simulation. (a) Receptor in the unbound form and (b) Receptor bound to eugenol. E is the final potential energy, E-p is the potential energy without the electrostatics component, T is the temperature, P is the pressure and V is the volume.
(TIFF)

S5 Fig Ligand-protein interactions during the 20 ns MD simulations of the eugenol-bound form of mouse OR73. The figure shows the fraction of number of snapshots where interactions between ligand and residues were retained at the binding site for the 20 ns MD simulation. Ser 113 is present more than 35% of the simulation time at the binding site.
(TIFF)

S6 Fig The residues present at the ligand-binding site for more than 35% of the total simulation time. Ser 113 is seen to be present at the binding site and forms a H-bond with the ligand for more than 35% of the total simulation time. The figure is obtained using the “Ligand Interaction Diagram” of the GLIDE software (**Schrödinger Release 2013–1**:, version 2.6, Schrödinger, LLC, New York, NY, 2013).
(TIFF)

S7 Fig The ligand-binding residues for the mouse OR-eugenol complex mapped on the three-dimensional homology model. The ligand-binding residues (red) of mouse OR-eugenol complex (blue) are spatially clustered and the ligand remains in this binding pocket throughout the MD simulations.
(TIFF)
