# Supplementary figures and images for: Computational Approaches for Decoding Select Odorant-Olfactory Receptor Interactions Using Mini-Virtual Screening
Source: PLoS One. 2015 Jul 29;10(7):e0131077. doi: 10.1371/journal.pone.0131077 (PMC4519343; doi:10.1371/journal.pone.0131077)

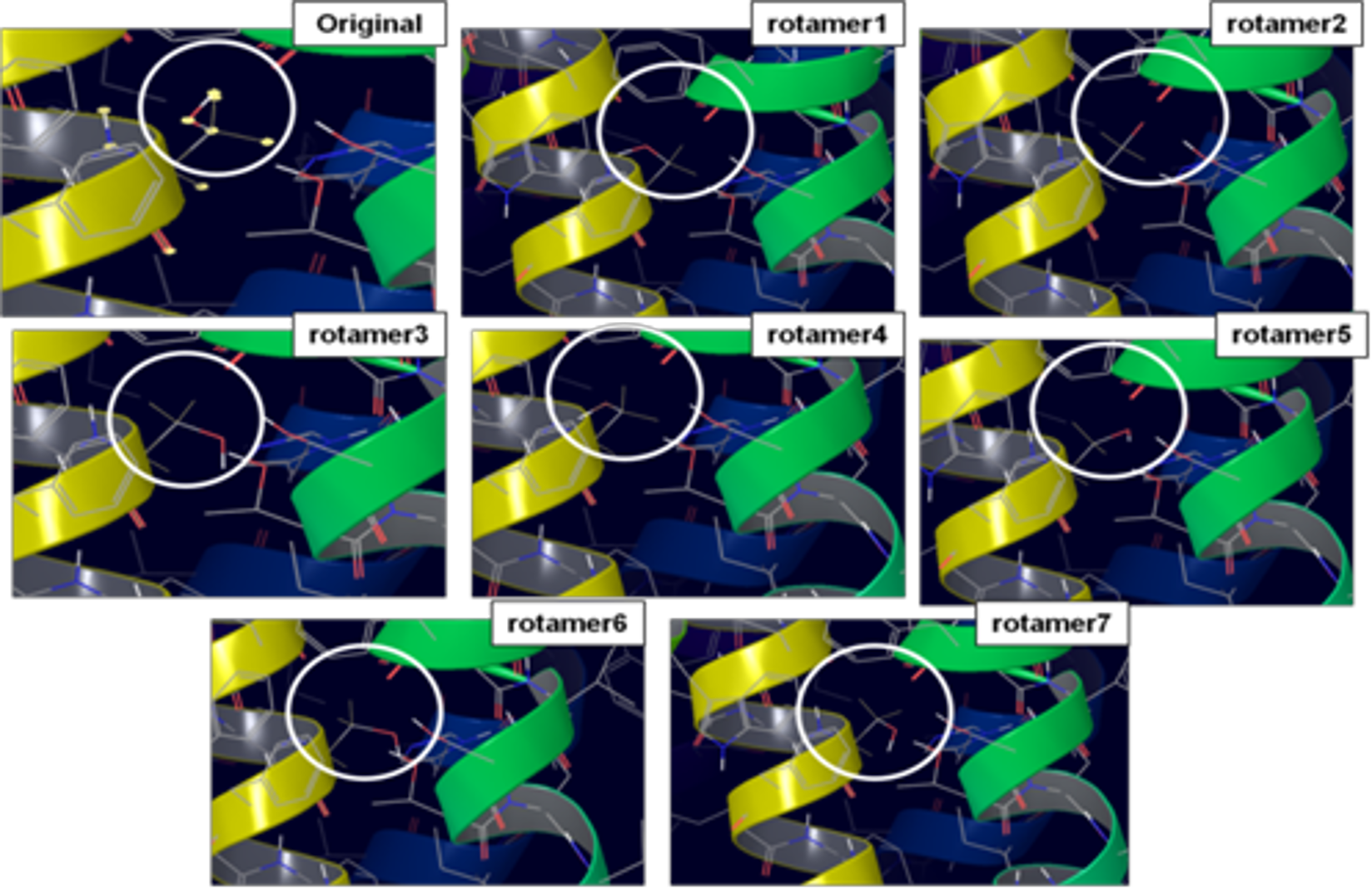

Supplement: S1 Fig — Different rotameric states of Ser 113 were obtained using PRIME module of GLIDE. Ser 113 was shown to be important for binding of eugenol to mOR-EG. Different rotameric states of Ser 113 were used to check if the odorants form any bonded interaction with Ser 113 in its different rotameric states. The change in rotameric state of Ser 113 residue did not increase its proximity to the ligand. The figure is obtained using PyMOL (The PyMOL Molecular Graphics System, Version 1.5.0.4 Schrödinger, LLC). (TIF) [file pone.0131077.s001.tif]

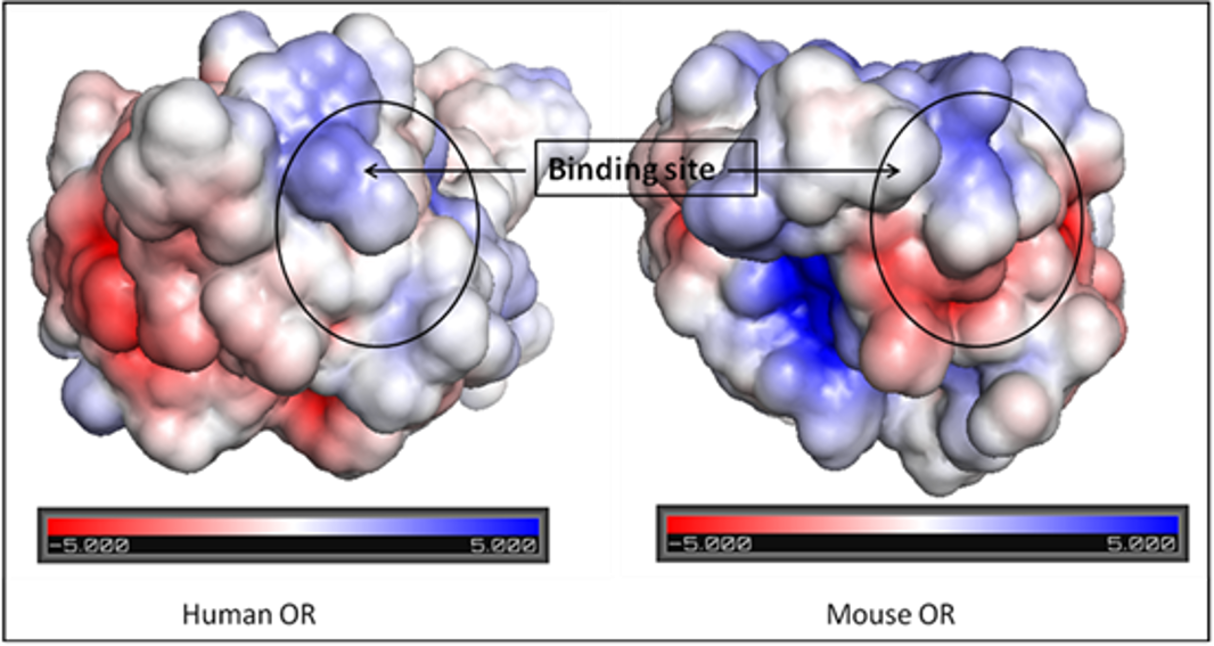

Supplement: S2 Fig — Electrostatics is represented by calculated charge from red (acidic residues; -5 kbT/ec) to blue (basic residues; +5 kbT/ec) as in Adaptive Poisson—Boltzmann Solver (APBS) program in PyMOL (The PyMOL Molecular Graphics System, Version 1.2r3pre, Schrödinger, LLC.). The surface electrostatics at the binding site of human and mouse ORs (Sequence identity is 84%) is different and this explains the difference in varied ligand profiles of these receptors. (TIFF) [file pone.0131077.s002.tiff]

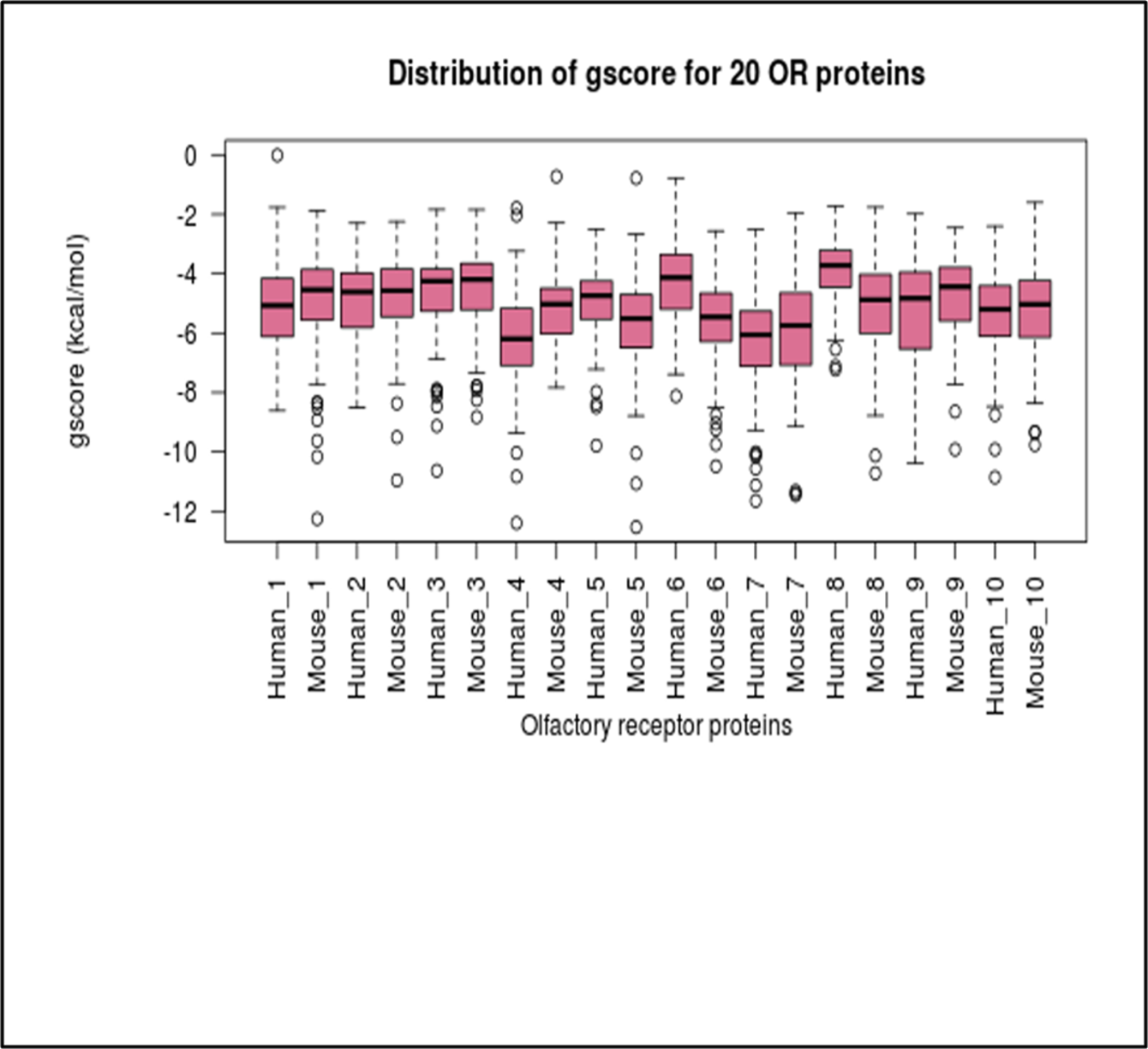

Supplement: S3 Fig — The distribution of gscores for OR proteins and the odor molecules has been represented as a Box-Whisker plot (prepared using R-scripts). The plot represents the spread of gscores for 125 odor molecules against each of the 20 OR proteins. The gscores range from -4 to -6 kcal/mol for all the ORs under study. For the OR pair (Pair 2) with highest sequence identity (84%) the median value of gscore is equal. The circles outside the plot represent the outliers. The OR proteins are numbered based on the pair they belong to. For eg: Human 1 and Mouse 1 belong to OR pair 1 under study. (TIFF) [file pone.0131077.s003.tiff]

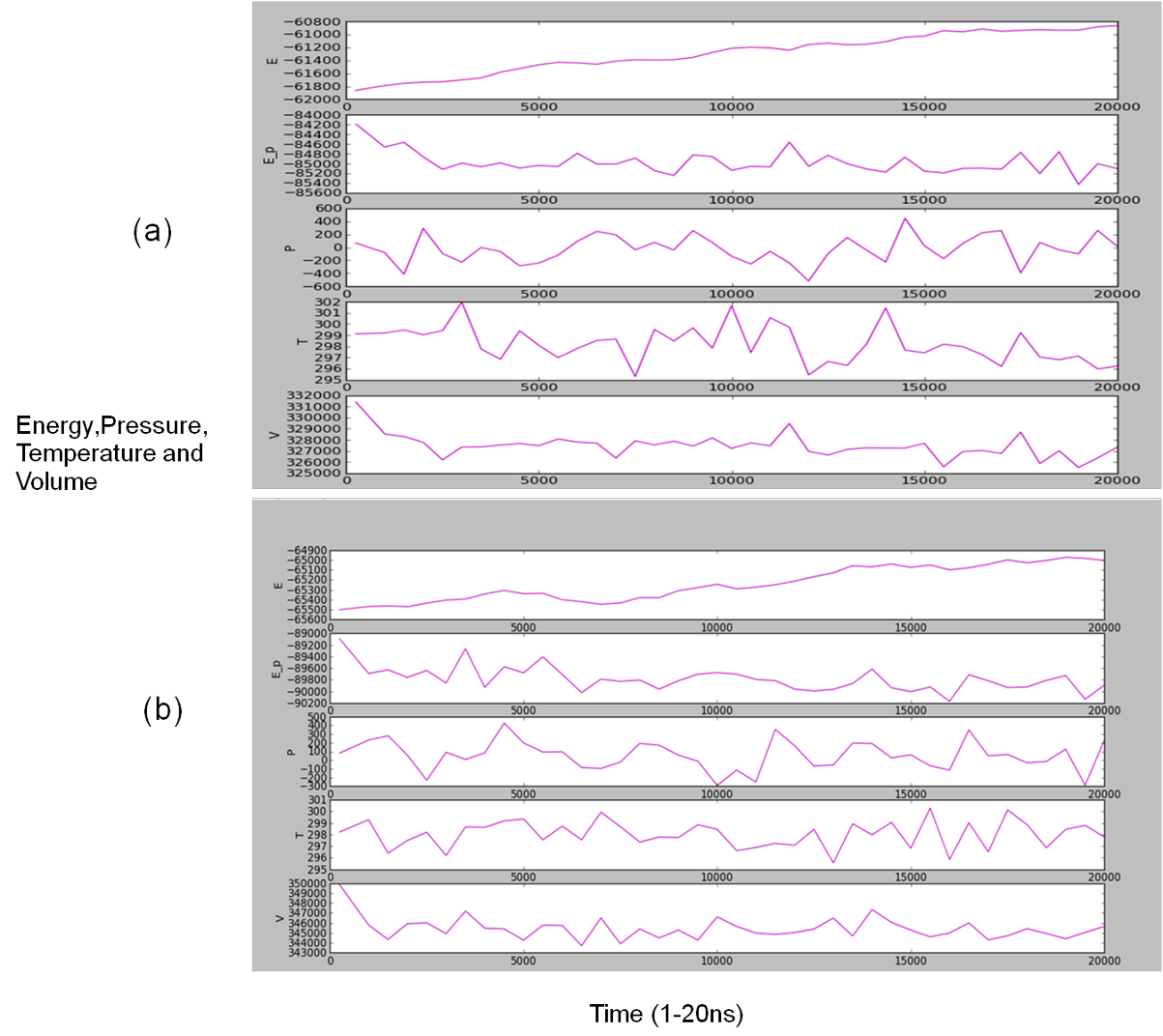

Supplement: S4 Fig — (a) Receptor in the unbound form and (b) Receptor bound to eugenol. E is the final potential energy, E-p is the potential energy without the electrostatics component, T is the temperature, P is the pressure and V is the volume. (TIFF) [file pone.0131077.s004.tiff]

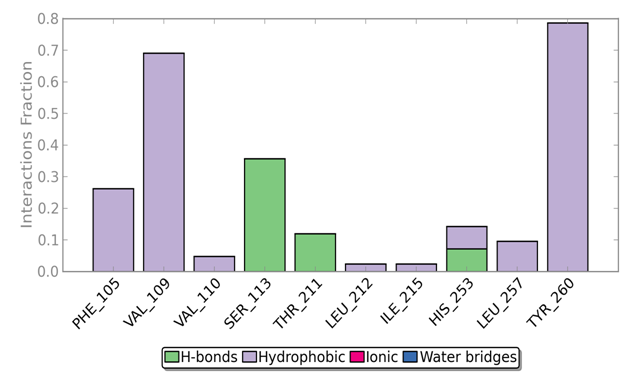

Supplement: S5 Fig — The figure shows the fraction of number of snapshots where interactions between ligand and residues were retained at the binding site for the 20 ns MD simulation. Ser 113 is present more than 35% of the simulation time at the binding site. (TIFF) [file pone.0131077.s005.tiff]

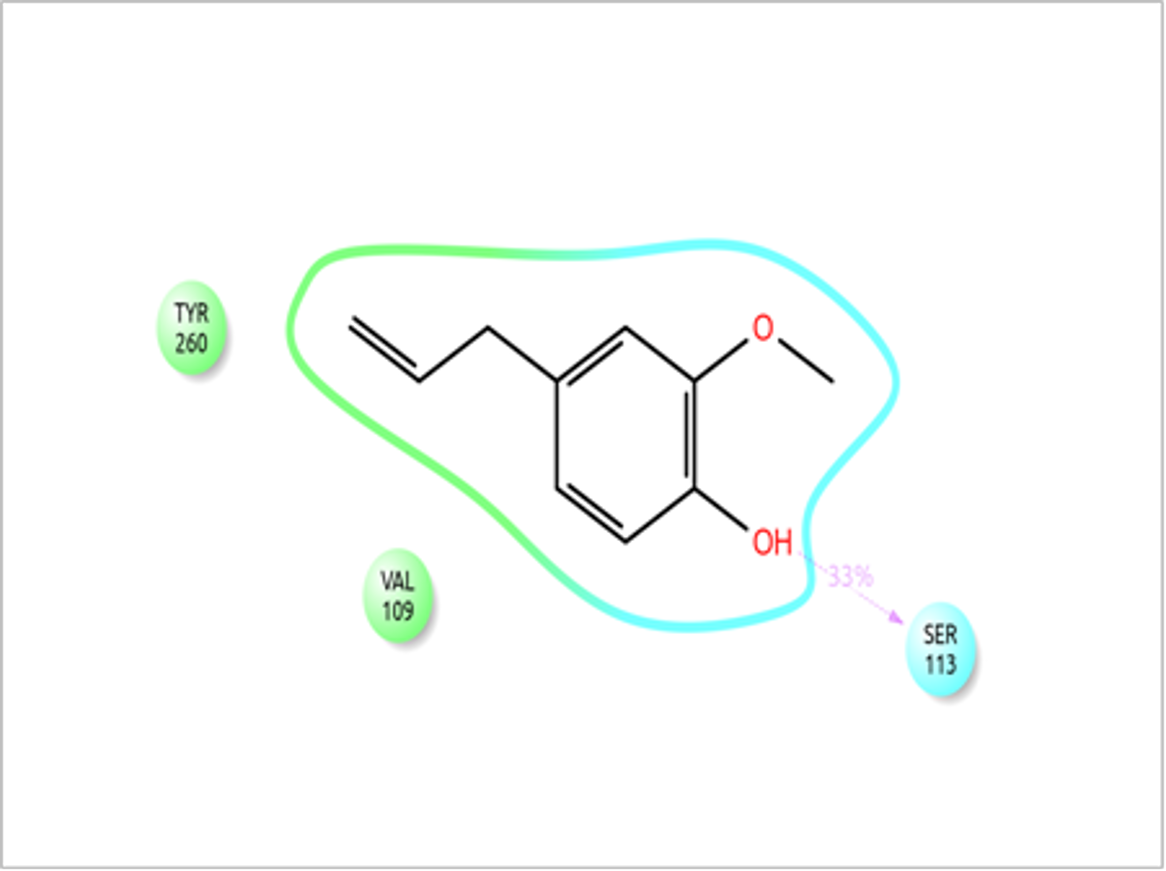

Supplement: S6 Fig — Ser 113 is seen to be present at the binding site and forms a H-bond with the ligand for more than 35% of the total simulation time. The figure is obtained using the “Ligand Interaction Diagram” of the GLIDE software (Schrödinger Release 2013–1:, version 2.6, Schrödinger, LLC, New York, NY, 2013). (TIFF) [file pone.0131077.s006.tiff]

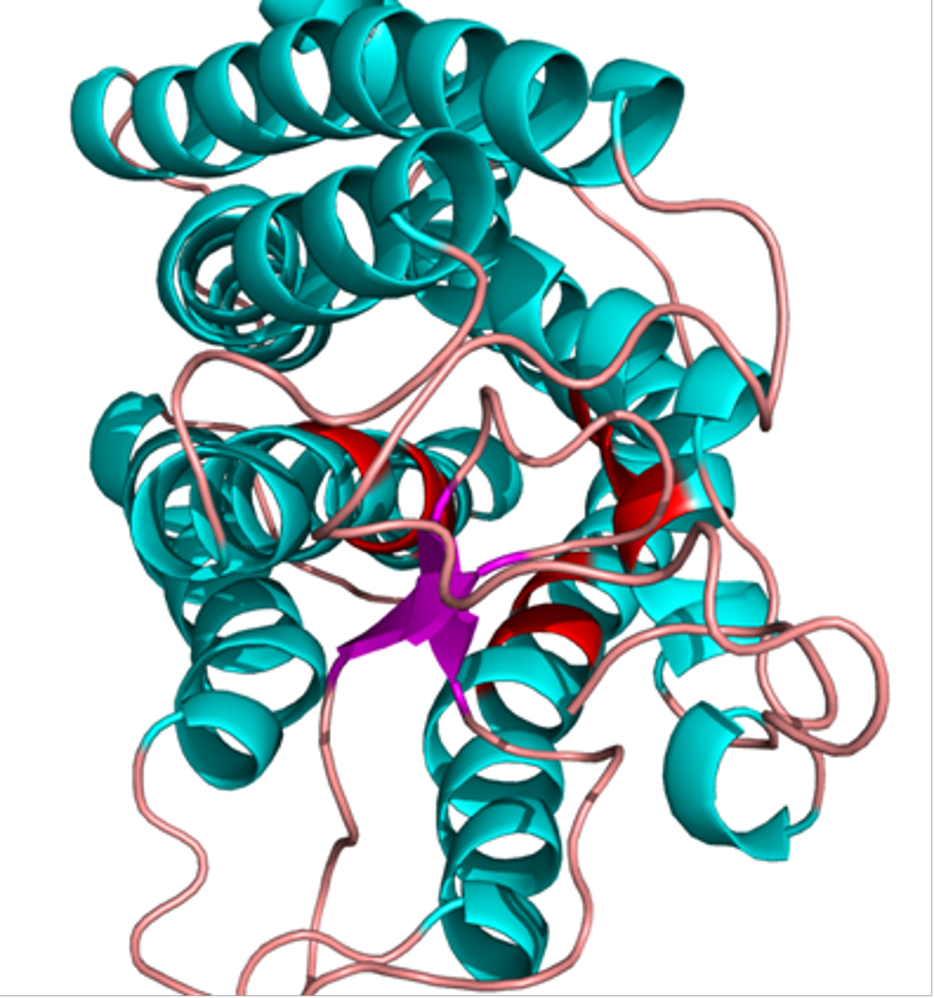

Supplement: S7 Fig — The ligand-binding residues (red) of mouse OR-eugenol complex (blue) are spatially clustered and the ligand remains in this binding pocket throughout the MD simulations. (TIFF) [file pone.0131077.s007.tiff]
